# Tibet plateau probiotic mitigates chromate toxicity in mice by alleviating oxidative stress in gut microbiota

**DOI:** 10.1038/s42003-020-0968-3

**Published:** 2020-05-15

**Authors:** Pengya Feng, Ze Ye, Huawen Han, Zhenmin Ling, Tuoyu Zhou, Shuai Zhao, Amanpreet Kaur Virk, Apurva Kakade, Abd El-Fatah Abomohra, Marwa M. El-Dalatony, EI-Sayed Salama, Pu Liu, Xiangkai Li

**Affiliations:** 1 Ministry of Education Key Laboratory of Cell Activities and Stress Adaptations, School of Life Science, Lanzhou University, Lanzhou, Gansu 730000 P. R. China; 2 Faculty of Applied Sciences and Biotechnology, Shoolini University of Biotechnology and Management Sciences, Solan, 173212 Himachal Pradesh India; 3 Botany Department, Faculty of Science, Tanta University, 31527 Tanta, Egypt; 4 Department of Earth Resources and Environmental Engineering, Hanyang University, Seoul, 04763 South Korea; 5 Department of Occupational and Environmental Health, School of Public Health, Lanzhou University, Lanzhou, Gansu 730000 P. R. China

**Keywords:** Microbiology, Gene expression analysis, Gene expression profiling, Immunological disorders, Microbial communities

## Abstract

Heavy metal contamination in food endangers human health. Probiotics can protect animals and human against heavy metals, but the detoxification mechanism has not been fully clarified. Here, mice were supplemented with *Pediococcus acidilactici* strain BT36 isolated from Tibetan plateau yogurt, with strong antioxidant activity but no chromate reduction ability for 20 days to ensure gut colonization. Strain BT36 decreased chromate accumulation, reduced oxidative stress, and attenuated histological damage in the liver of mice. 16S rRNA and metatranscriptome sequencing analysis of fecal microbiota showed that BT36 reversed Cr(VI)-induced changes in gut microbial composition and metabolic activity. Specifically, BT36 recovered the expressions of 788 genes, including 34 inherent Cr remediation-relevant genes. Functional analysis of 10 unannotated genes regulated by BT36 suggested the existence of a new Cr(VI)-reduction gene in the gut microbiota. Thus, BT36 can modulate the gut microbiota in response to Cr(VI) induced oxidative stress and protect against Cr toxicity.

## Abstract (web summary)

Feng et al. show that colonization of mice with *Pediococcus acidilactici* strain BT36 reduces chromate accumulation in the liver, which is associated with changes in expression of a Cr(VI)-resistance gene in the gut microbiota. This study suggests that BT36 regulates the gut microbiota in response to chrome exposure, protecting mice against chromate toxicity.

## Introduction

With global expansion of industrial activities, heavy metals (HMs) are increasingly released to environment through various anthropogenic processes, leading to contamination of the atmosphere, water, and soil. Up to 2.5 million potentially contaminated sites were reported in Europe^1^. In the Campine region of Belgium and the Netherlands, 700 km^2^ of land have been polluted by atmospheric deposition of cadmium (Cd), zinc, and lead (Pb)^2^. In China alone, 26.67 million hm^2^ of cultivated land has been contaminated with HMs with various degrees, accounting for 20% of the total arable land^3^. HMs can be accumulated in crops and eventually transferred to human body via dietary intake^4^. In recent years, many technologies such as phytoremediation, physical-chemical remediation and biological approaches have been proposed to cope with soil HM contamination. Phytoremediation has been generally accepted for decontamination of HMs-polluted soils compared with conventional physical-chemical remediation, due to its cost-effective, environment- and eco-friendly on-site application^5^. However, it may be less effective considering the vast area of pollution and the long remediation duration required for plant growth^6^. In the near future, humans will be inevitably exposed to HM contaminated foods, especially in developing countries. Hence, it is urgent to seek alternative and practical ways to protect humans from HMs.

Mounting evidences suggested that several lactobacilli and potential probiotics might be useful for detoxification of ingested HMs in both human and murine models. A pilot study in an at-risk human population in Tanzania demonstrated the potential of *Lactobacillus rhamnosus* GR-1 supplemented yogurt in lowering the blood levels of toxic metals^7^. Similarly, several *L. plantarum* strains were found effective in mitigating the toxic effects of ingested cadmium, lead, and chromate in mice via multiple routes, mainly by intestinal sequestration and intestinal barrier protection^6,8,9^. However, as indicated in recent reports, the number of probiotic strains that colonize the gastrointestinal tract is extremely limited, and thus, it is unknown how the restricted number of probiotics exert their influence to achieve protective effects against HM pollutants.

Gut microbiota, multispecies community of microbes that resides in the gastrointestinal tract, is considered the first line of defense against ingested environmental xenobiotics^10^. It plays an important role in host health by metabolizing an array of dietary and host-derived compounds and by processing HMs as well^11^. The “barrier” function of the GM in limiting HM biosorption has been demonstrated by contrasting germ-free and conventional mice^12^. A recent metatranscriptomic study on intestinal microorganisms provided evidence that HM-detoxifying genes such as glutathione (GSH) are present and active in the genome of gut microbes^13^. Further, HM stress could alter the composition and possibly the antioxidant function of the GM^13,14^. However, the influence of probiotics on the intestinal microorganisms in mediating the detoxification of HMs is unclear.

Chromium (Cr) is an omnipresent environmental toxic contaminant and is mainly present in trivalent (III) or hexavalent (VI) forms^15^. Cr(VI) can be easily absorbed by living cells and is a highly hazardous form that causes cancer and other health problems^16^. Cr(VI) can be reduced to Cr(III), which is poorly absorbed, less toxic, and considered an essential trace element for humans^17^. In a previous study, an *L. plantarum* strain with strong Cr(VI)-reducing ability effectively diminished Cr accumulation in mouse tissues and enhanced the Cr(VI)-reducing ability of fecal microbes^6^. Hence, we hypothesize that the inherent Cr(VI)-detoxifying gene activity of commensal intestinal microorganisms is repressed under Cr(VI) stress, and could be recovered by some probiotics, resulting in enhanced capacity of the GM to combat Cr(VI). A probiotic candidate strain *Pediococcus acidilactici* BT36 (BT36), with antioxidant but no Cr(VI)-reducing capacity, isolated from dairy products of the Tibetan plateau of China, was selected as a Cr(VI)-detoxifying agent in mice, allowing us to gain insights into the underlying mechanisms of probiotics and gut microorganisms in integrated HM detoxification.

## Results

### BT36 reduced Cr accumulation in tissues

**Fig. 1:**
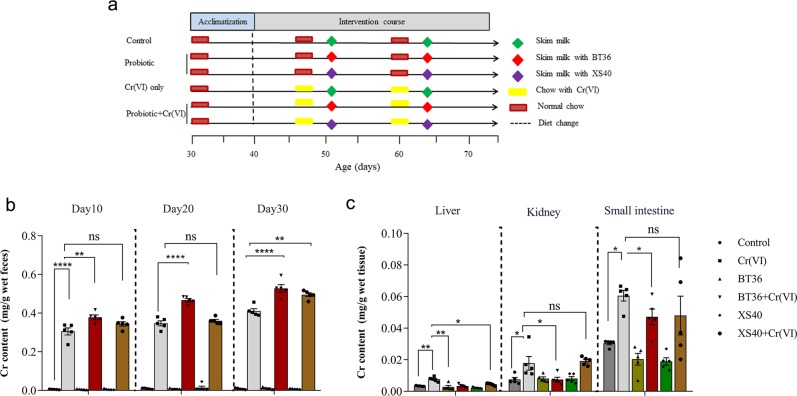
Effects of BT36 on the excrement of Cr and Cr accumulation in tissues. **a** Experimental grouping. **b** Effects of BT36 and XS40 treatments on Cr levels in feces. **c** Effects of BT36 and XS40 treatments on Cr levels in tissues of mice. Values are expressed as mean ± SEM (*n* = 5). Statistical analyses were conducted using the the one-way ANOVA followed by Tukey’s post hoc test. *p < 0.05, **p < 0.01, ***p < 0.001, ****p < 0.0001; ns: not significant.

BT36 mildly attenuated Cr(VI)-induced body weight changes in mice and no significant differences in food consumption were observed among the six groups (Supplementary Fig. 1), suggesting that the discrepancy in growth rate resulted from Cr(VI) treatment rather than food consumption. Cr level in the feces of the Cr(VI) group was markedly higher than that in the control group, increasing from 0.0056 ± 0.0021 mg/g to 0.3055 ± 0.06872 mg/g at day 10 (*p* < 0.0001), and the level increased progressively with time. With BT36 intervention, the Cr content in the feces was further increased on day 10 (0.3785 ± 0.0356 mg/g wet feces, *p* = 0.0034), day 20 (0.4667 ± 0.0305 mg/g, *p* < 0.001) and day 30 (0.5278 ± 0.0577 mg/g, *p* < 0.001). In comparison, the promoting effect of strain *P. acidilactici* XS40, which presented a lower Cr(VI)-resisting and antioxidation capacity than BT36 (Supplementary Fig. 2), was only observed on day 30 (0.4953 ± 0.0350 mg/g, *p* = 0.0046) (Fig. 1b).

Intake of Cr-added chow caused increased Cr(VI) accumulation in the liver (0.0076 ± 0.0017 mg/g, *p* = 0.0064), kidney (0.0178 ± 0.0043 mg/g, *p* = 0.0229) and small intestine (0.0606 ± 0.013 mg/g, *p* = 0.0150) (Fig. 1c). In the Cr(VI) + BT36 group, the amount of residual Cr decreased to 54.80% (liver, *p* = 0.0015), 57.00% (kidney, *p* = 0.0219), and 22.12% (small intestine, *p* = 0.0491), compared with that in the Cr(VI) group, whereas strain XS40 reduced the Cr level only in the liver (*p* = 0.0209) and hardly in the kidney or small intestine. These results showed that Cr(VI) intake increased the Cr levels in tissues and feces, and that supplementation with BT36 rather than XS40, effectively reduced Cr accumulation and facilitated its excretion.

### BT36 alleviated Cr(VI)-induced damage in tissues

**Fig. 2:**
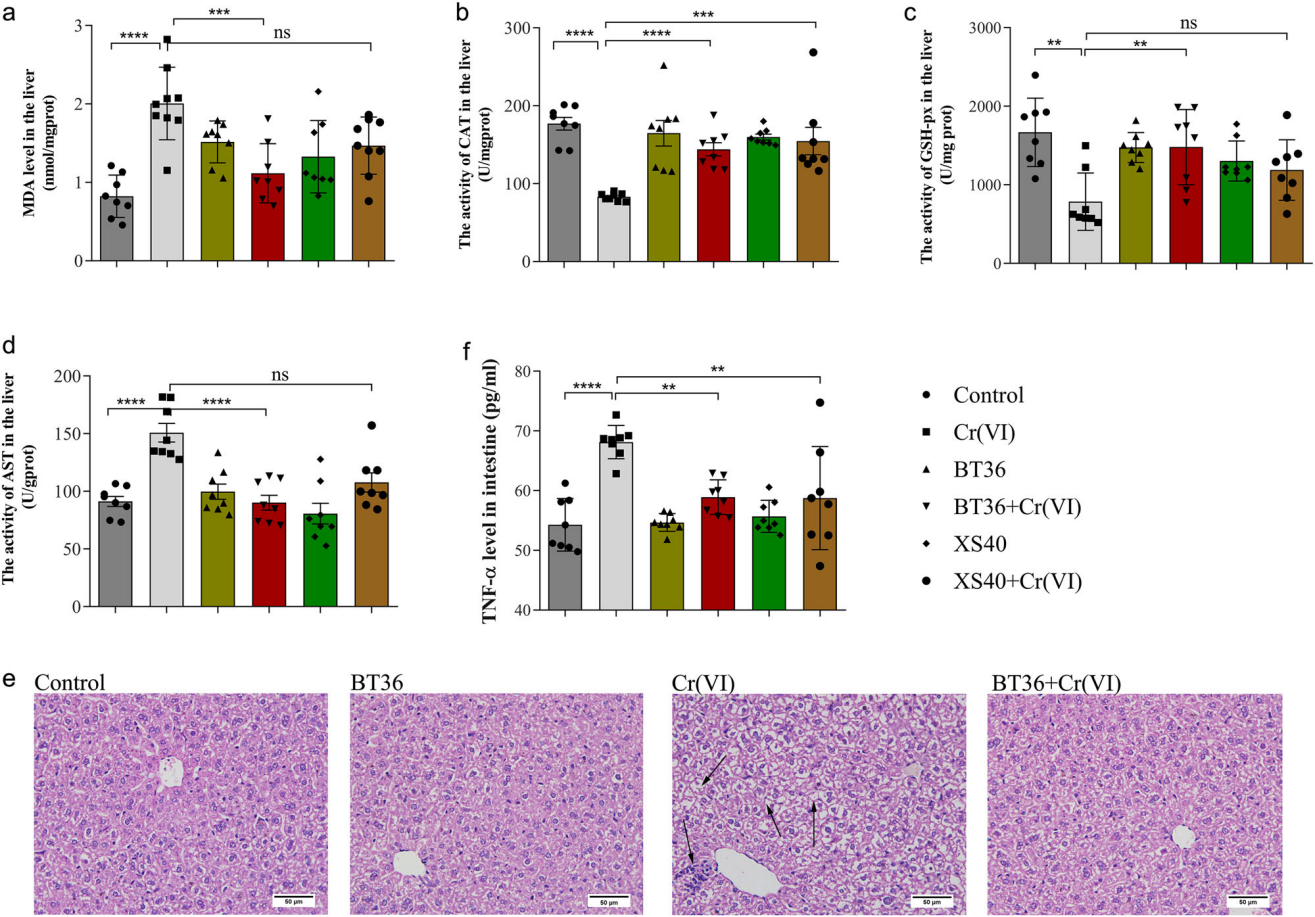
Effects of BT36 and XS40 on Cr(VI)-induced hepatic and small intestinal damage in mice. MDA level (**a**), CAT (**b**), GSH-px (**c**), and AST activities (**d**), histological analysis of liver under a microscope (**e**, 600×) and TNF-α level of small intestine (**f**) are shown. For **a**–**d**, **f**, values are expressed as the mean ± SEM (*n* = 8). Statistical analyses were conducted using the one-way ANOVA followed by Tukey’s post hoc test. *p < 0.05, **p < 0.01, ***p < 0.001, ****p < 0.0001; ns: not significant. Arrows in figure **e** indicate the sites of liver damage and scal**e** bars: 50 μm.

Metal toxicity is closely associated with increased oxidative stress in tissues. The present in vitro study indicated that BT36 presented robust antioxidant ability by scavenging 89.84% of hydroxyl radicals and 86.88% of 1,1-Diphenyl-2-picrylhydrazyl (DPPH) radicals (Supplementary Fig. 2b). The antioxidant effect of BT36 on the liver was analyzed by measuring the levels of MDA, CAT, and GSH-px. Cr ingestion caused hepatic oxidative stress indicated by a higher level of MDA (*p* < 0.0001) and lower levels of CAT (*p* < 0.0001) and GSH-px (*p* = 0.0003) activities. Co-administration of BT36 significantly reversed the Cr-induced changes in all the above oxidative stress markers (MDA, *p* = 0.0002; CAT, *p* = 0.0044; GSH-px, *p* = 0.0058) (Fig. 2a). XS40 only restored the CAT level (*p* = 0.0006) (Fig. 2b, c), indicating BT36 was more effective than XS40 in alleviating Cr(VI)-induced hepatic oxidative stress. To evaluate the hepatic damages in different conditions, both biochemical and histological analyses were performed. Compared with the control group (91.2230 ± 17.8892 U/g protein), AST activity was significantly increased after exposure to Cr(VI) (150.8226 ± 23.1181 U/g protein, *p* < 0.0001), which was remarkably reversed by BT36 treatment (90.2475 ± 19.7306 U/g protein, *p* < 0.0001) but not XS40 (Fig. 2d). As shown in Fig. 2e, Cr(VI) exposure caused marked liver injury including cytoplasmic vacuolization, pyknotic nuclei, and chromatin condensation, which was attenuated by BT36 administration. In addition, paraffin sections of the liver tissue from BT36-only mice revealed no signs of histological damage, implying that BT36 had no detrimental effects on the hepatic tissue in mice. Moreover, both BT36 and XS40 significantly decreased the levels of TNF-α in the small intestine induced by Cr(VI) (BT36, *p* = 0.0022; XS40, *p* = 0.0018) (Fig. 2f), suggesting that BT36 and XS40 were effective in mitigating Cr(VI)-induced inflammation.

### BT36 increased the Cr(VI)-reduction capacity of GM

**Fig. 3:**
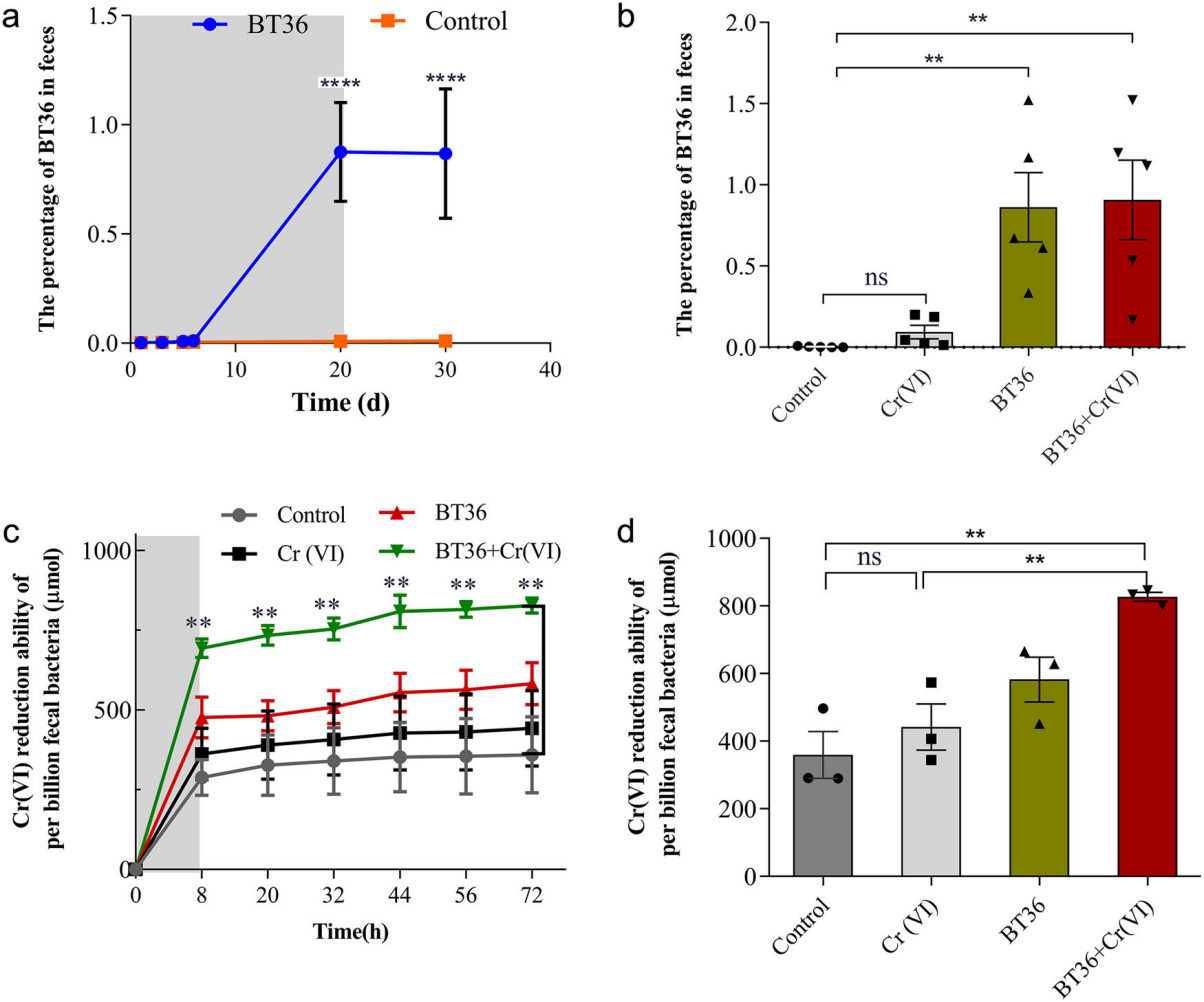
Effects of BT36 on the Cr(VI)-reduction capacity of gut microbiota. Quantification of BT36 in feces during BT36-only treatment (**a**) and among four groups on treatment day 20 (**b**) (n = 5). The Cr(VI) reduction ability of cultivatable fecal bacteria after 72 h (**c**) and 44 h (**d**) of culture under anaerobic conditions at 37 °C (*n* = 3). The percentage of BT36 refers to the ratio of the BT36 count to the total bacterial number in feces. The bars in (**c**) and (**d**) represent the reduction ability of 10^9^ fecal bacteria. Values are expressed as the mean ± SEM. For **a**, statistical analyses were conducted using student’s *t*-test. For **b**, **c** and **d**, statistical analyses were conducted using the one-way ANOVA followed by Tukey’s post hoc test. *p < 0.05, **p < 0.01, ***p < 0.001, ****p < 0.0001; ns: not significant.

As probiotic strain expansion in stool is considered a sign of probiotic efficacy^18–20^, the abundance of BT36 was assessed by quantifying the copy number of the ldhD gene in fecal DNA by RT-qPCR at different time points (Fig. 3a). The relative abundance of BT36 was significantly higher on day 20 (0.8752%, *p* < 0.0001) and day 30 (0.8685%, *p* < 0.0001) in mice supplemented with BT36 than in the control groups (0.0080%) (Fig. 3a), and co-treatment with Cr(VI) and BT36 also significantly increased the abundance of BT36 compared with control group (*p* = 0.0060) (Fig. 3b), strongly suggesting that BT36 colonized the intestine regardless of Cr(VI) presence. Further analysis showed that colonization of BT36 elevated the reduction rate of Cr(VI) (69.39%) in anaerobic culture of fecal microbes, compared with the Cr(VI) only treatment (36.19%) (*p* = 0.0062) and control group (28.80%) (*p* = 0.0021) at day 44 (Fig. 3c, d). In the BT36-only group, the Cr(VI) reduction ability of fecal microbes (47.65%) was also increased compared with both the control group (*p* = 0.1320) and Cr(VI) group (*p* = 0.4376), but with no significant difference.

### BT36 regulated Cr(VI)-induced GM dysbiosis in mice

**Fig. 4:**
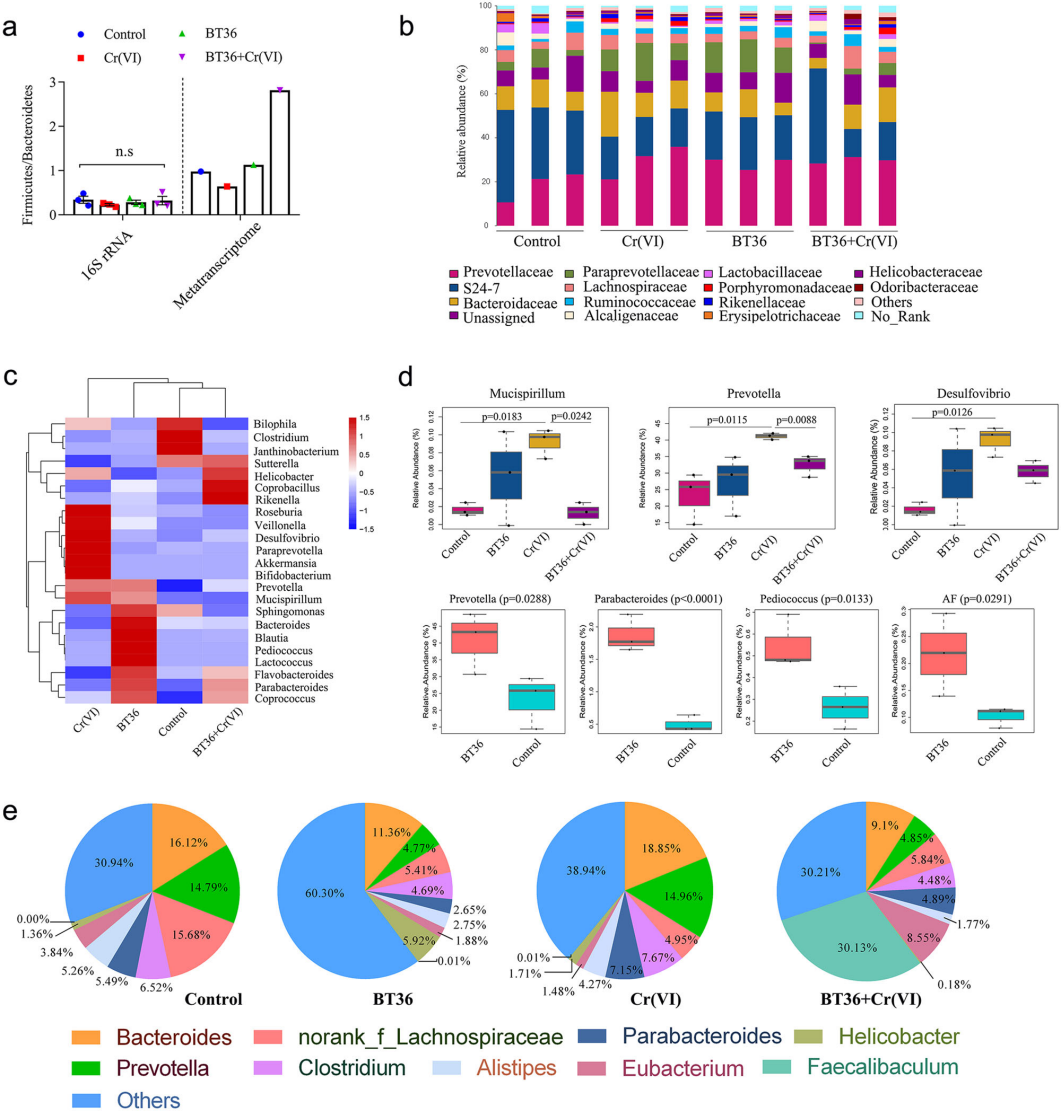
Composition of total bacteria (16S rRNA) and metabolically active bacteria (metatranscriptome). **a** The ratio of Firmicutes and Bacteroidetes. For 16S rRNA analysis, statistical analyses were conducted using the one-way ANOVA followed by Tukey’s post hoc test (*n* = 3). **b** Relative abundance (% of total reads) of bacterial 16S rRNA gene at the family level (*n* = 3). **c** Heatmap of the genus based on 16S rRNA sequencing (*n* = 3). The color of each rectangle in the heatmap reprensents the normalized and log-transformed relative abundance of each genus in four groups by a gradient of color from blue (low abundance, −1.5) to red (high abundance, 1.5). **d** Alterations in genus with statistically significant differences based on 16S rRNA sequencing (*n* = 3). The box- plots show the maximum, minimum and median, and the whisker indicates total range. **e** Taxonomic assignment of mRNA in each sample at the genus level. By default, all species whose expression abundance was less than 1% in all samples were combined with others. For **d**, STAMP v2.0.0 software (*p* < 0.05) was employed to analyze statistical significance between the fecal microbiome of four groups using ANOVA with Benjamini-Hochberg FDR multiple test correction, and to analyze statistical significance between the fecal microbiome of two groups using one sided *t*-test.

Both HMs and probiotics can impact the composition of the GM based on 16S rRNA sequencing results. The sequencing of gut microbial 16S rRNA from four groups (*n* = 12) resulted in a total of 1,263,860 reads. After quality filtering, 1,150,012 (min: 28,677; max: 290,528; mean: 95,834; median: 70,228) reads remained for the assignment of ASVs. Overall, 344,124 reads from 12 samples (average of 28,677 reads per sample) on the rarified data set were used for the further analysis. The Shannon and Chao richness estimator (Supplementary Table 1) showed that the diversity of the total bacterial community was stable among different groups. At the phylum level, the ratio of *Firmicutes* and *Bacteroidetes* (F/B) showed no variation among groups (Fig. 4a). The most abundant families were S24-7 (18.24–34.53%), *Prevotellaceae* (18.39–29.74%), and *Bacteroidaceae* (9.01–14.68%), followed by *Lachnospiraceae* (3.93–6.12%) and *Paraprevotellaceae* (2.88–13.45%) (Fig. 4b). The genera heatmap showed alterations with different treatments (Fig. 4c). Cluster analysis indicated that the structure of gut microbiota in the BT36 plus Cr(VI) group was closer to the control than the Cr(VI) group, suggesting alleviation in Cr(VI) toxicity by BT36. Based on the significance analysis of genera using STAMP software, Cr(VI) exposure induced a significant increase in the abundance of *Mucispirillum* (*p* = 0.0183), *Desulfovibrio* (*p* = 0.0126), and *Prevotella* (*p* = 0.0115) compared with the control. *Mucispirillum* (*p* = 0.0242), and *Prevotella* (*p* = 0.0088) were significantly restored with BT36 treatment (Fig. 4d). In the BT36-only group, the relative abundance of *Parabacteroides* (*p* < 0.0001), *Pediococcus* (*p* = 0.0133), *Prevotella* (*p* = 0.0288) and AF12 (*p* = 0.0291) was significantly higher than that in the control group (Fig. 4d). Notably, detection of the genus *Pediococcus* in the BT36-only group confirmed the results of BT36 colonization by RT-PCR (Fig. 3a), and failure to identify *Pediococcus* in the BT36 + Cr(VI) group might have resulted from the insufficient sensitivity of 16S rRNA sequencing.

Metatranscriptomic sequencing was performed to further identify the metabolically active bacteria in feces. On average, 24 million raw sequence reads were obtained from the metatranscriptome of the four samples. After removing the adapters and low-quality reads, 60,546,020 clean reads with 8.17 G clean bases, 55,070,192 clean reads with 7.47 G clean bases, 44,050,042 cleans reads with 5.89 G clean bases and 40,869,144 clean reads with 5.46 G clean bases were obtained from BT36, BT36 + Cr(VI), control and Cr(VI) group, respectively. The results indicated that the amount of data met the quality requirements for subsequent analysis. The results of the de novo assembly were shown in Supplementary Table 2. We obtained 14,659, 78,611, 87,739 and 92,503 unigenes with the N50 (N90) length of 556 bp (329 bp), 813 bp (347 bp), 945 bp (358 bp), 738 bp (344 bp) from BT36, BT36 + Cr(VI), control and Cr(VI) group, respectively. There were most unigenes with length of 1–700 bp in all samples (Supplementary Fig. 3). Exposure to Cr(VI) remarkably decreased F/B from 0.98 to 0.64 compared with the control group, whereas BT36 reversed the ratio toward the control level and even higher (2.81) (Fig. 4a and Supplementary Table 3). At the genus level (Fig. 4e), Cr(VI) exposure altered the structure of GM, specifically, the relative abundances of *Bacteroides*, *Clostridium*, *Parabacteroides*, and *Helicobacter* were increased, and the relative abundance of no_rank_*Lachnospiraceae* and *Eubacterium* were reduced. After BT36 intervention, the abundances of *Eubacterium* (from 1.48 to 8.55%), norank_f_*Lachnospiraceae* (4.95 to 5.84%), *Helicobacter* (from 1.71 to 0.18%), *Parabacteroides* (from 7.15 to 4.89%), and *Clostridium* (from 7.67 to 4.48%) were either fully or partially restored. All these results indicate that BT36 has a restorative effect on GM.

### BT36 upregulated DEGs of GM relevant to Cr(VI) remediation

**Fig. 5:**
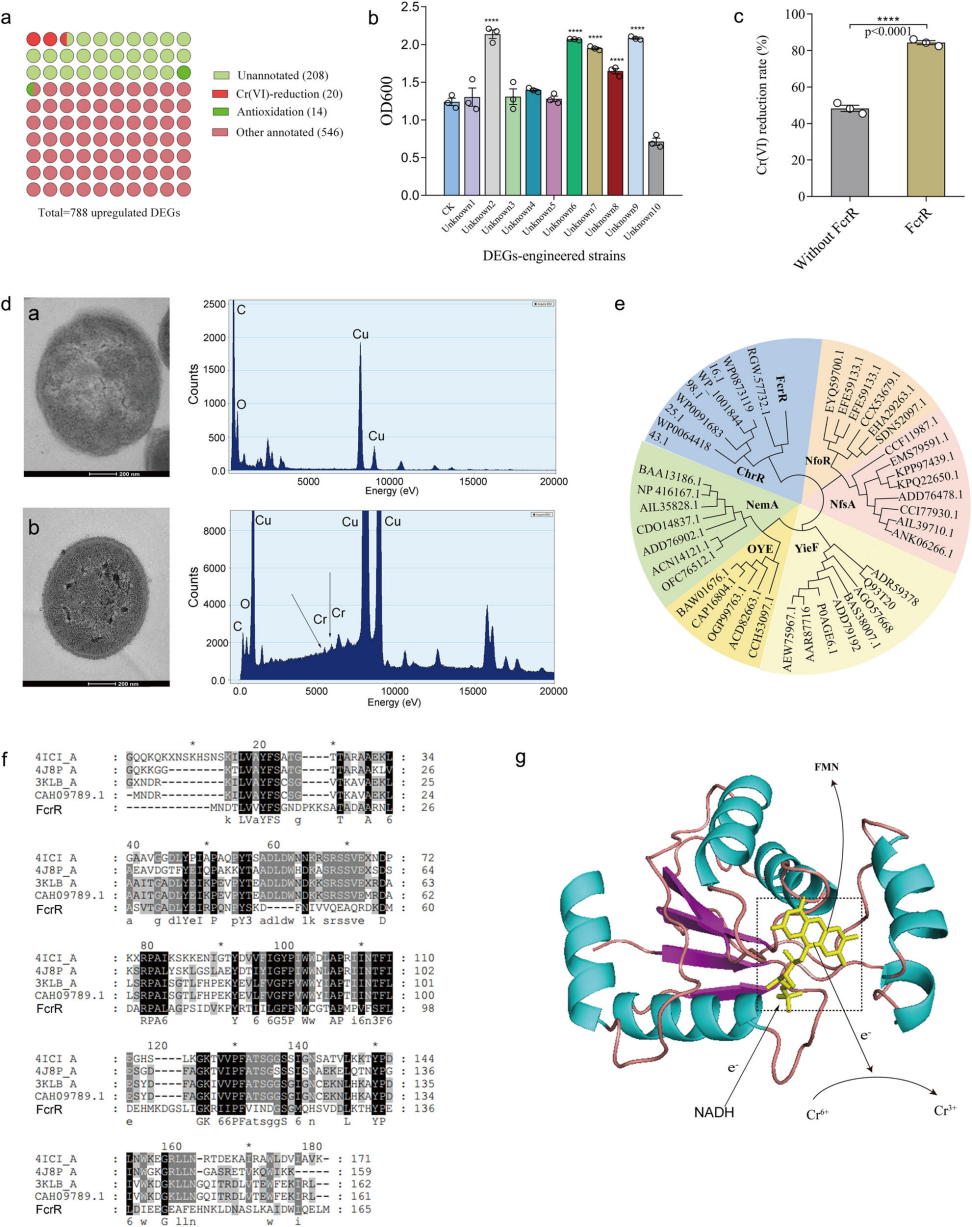
Functional analysis of selected unannotated DEGs. **a** The distribution of Cr remediation relevant genes in total DEGs. **b** The growth of engineered *E*. *coli* strains harboring DEGs in LB medium with 1 mM Cr(VI) (*n* = 3). CK represents *E. coli* BL21 containing the empty plasmid pET28a (+), unknown 1–10 represents *E. coli* BL21 with pET28a harboring gene *comp53346*, *53095*, *24337*, *53087*, *53032*, *51796*, *52516*, *53047*, *53431*, and *53299*, respectively. **c** Cr(VI) reduction rate of engineered *E*. *coli* BL21 with *53431* (*n* = 3). **d** TEM and EDX analyses of *E. coli* FcrR after inoculated in LB medium and incubated overnight without Cr(VI) or with 1 mM Cr(VI), and the corresponding EDX spectra. Arrows show intracellular Cr(III) precipitates, and scale bars: 200 nm. **e** Evolutionary relationship of FcrR with known chromate reductases from different genera. A composite evolutionary tree with the FcrR was generated using the Neighbor-Joining method based on the reported chromate reductase families (NfoR, NfsA, YieF, OYE, NemA, and ChrR). **f** Sequence analysis of FcrR homologs. The highly conserved residues are highlighted in black, and other residues with high levels of similarity are highlighted in gray. **g** Structural modeling of FcrR. For **b**, data significance was analyzed using the one-way ANOVA followed by Tukey’s post hoc test. For **c**, data significance was analyzed using student’s *t*-test. Significance was marked as *p < 0.05, **p < 0.01, ***p < 0.001, and ****p < 0.0001.

To screen for DEGs that might be involved in Cr(VI) detoxification, we used “log_2_FC(BT36 + Cr(VI)/Cr(VI)) ≥ 1” as the selecting criteria to identify BT36-regulating genes. In total, 788 DEGs whose expression was upregulated in BT36 + Cr(VI) were screened from 57,248 unigenes, including 20 reported Cr(VI)-reduction related genes, 14 known antioxidation related genes, 546 other annotated genes, and 208 hypothetical/function-unknown genes (Fig. 5a). The DEGs related to Cr(VI) reduction included thioredoxin, desulfoferrodoxin, flavodoxin, flavodoxin reductase, and FAD/NAD(P)-binding oxidoreductase. The DEGs associated with oxidative stress defense mainly included rubrerythrin family proteins, thiol peroxidase, rubredoxin, peroxiredoxin, SOD, and CAT (Supplementary Data 1 and Supplementary Fig. 4). To rule out the possibility that the 34 upregulated DEGs (Cr(VI)-reduction and antioxidation related) were expressed by BT36, the draft genome of BT36 was further sequenced and compared with the sequences of the 34 DEGs. All the DEGs were excluded from the probiotic BT36 by sequence alignment, indicating that all the DEGs were derived from the native GM. To evaluate the metatranscriptomics results, the expression of DEGs genes was determined by qRT-PCR. The upregulated DGEs *comp53028, 53032* and *53086* were randomly selected for qPCR verification, whose transcript in BT36 + Cr(VI) group were average 712.416, 245.242, and 226.731 times than that in Cr(VI) group, respectively (Supplementary Fig. 5). However, the selected genes (Ct value over 38) almost cannot be detect in the Cr(VI) and control group. The results were in accordance with the tendency of those observed in the metatranscriptomes analysis.

In addition to the annotated DEGs, unannotated DEGs are also important components of the metabolic functions in the GM. Thus, 10 upregulated genes with unknown function that met the criteria “log_2_FC(BT36 + Cr(VI)/Cr(VI)) ≥ 5” were randomly selected from the 208 function-unknown DEGs to test their Cr(VI) resistance in engineered *E. coli* (Fig. 5a). Strain unknown 2, 6, 7, 8, and 9 were more resistant to Cr(VI) than the control strain (*E. coli* harboring an empty pET28a) (Fig. 5b), suggesting that these upregulated function-unknown genes might contribute to Cr(VI) resistance in the GM. Amongst these, strain unknown 9 with corresponding gene *53431* reduced 84.37% of 1 mM Cr(VI) within 6 h of incubation, whereas the control strain reduced about 48.29% of Cr(VI) over the same time (Fig. 5c). Henceforth, the protein encoded by gene *53431* from the known intestinal *Faecalibaculum rodentium* was named FcrR, to indicate a Gut Cr(VI) Reductase. TEM analysis was conducted to locate the reduced Cr(VI) in *E. coli* FcrR. The TEM images clearly showed the formation of precipitates inside cells (Fig. 5d), whereas no precipitates were observed in the negative control without Cr(VI) treatment. The EDX results confirmed that the precipitates consisted of the chromium element, suggesting that Cr(VI) was reduced to Cr(III) inside the cells of strain FcrR. Collectively, these results demonstrated the presence of numerous unannotated genes in the GM that were Cr(VI)-resistant or able to reduce/detoxify Cr(VI) effectively, which might thus play roles in resisting Cr(VI) to maintain the homeostasis of GM and to protect the host.

To confirm the correlation between gene FcrR and other known chromate reductases, amino acid sequence alignment was performed to construct the phylogenetic evolutionary tree of FcrR and six classes of reported chromate reductase (NfoR, NfsA, YieF, OYE, NemA, and ChrR)^21–24^ (Fig. 5e). The FcrR (a putative chromate reductase) was shown to belong to clade ChrR and separate from the known chromate reductases. Furthermore, the amino acid sequence of FcrR was paired with multi-typical target sequences (*Bacteroides eggerthii* 4ICI_A, *Bacteroides uniformis* PDB 4J8P_A, *Bacteroides fragilis* 3KLB_A and *Bacteroides fragilis* CAH09789.1) using Clustal X, which all encode flavoproteins (Fig. 5f). The sequence identities of FcrR with 4ICI_A, 4J8P_A, 3KLB_A, and CAH09789.1 were 33.12%, 29.09%, 32.87%, and 33.56%, respectively. The secondary structure of FcrR was predicted using I-TASSER, showing 5 α-helices and 4 β-sheets. The structural model of FcrR was overlapped with the crystal structure template of a putative flavoprotein (PDB code 3KLB), suggesting that it also binds an FMN co-factor (Fig. 5g). The results revealed that protein FcrR was highly similar in the conserved region with the known flavoprotein 3KLB, based on sequence and structural analyses.

## Discussion

Oxidative stress is one of the key features of HM toxicity^25^. Imbalance between the production of free radicals and generation of antioxidants caused by oxidative stress causes damage to important biomolecules and cells, with potential impact on the whole organism^26^. In this study, exposure to Cr(VI) induced oxidative stress in the liver of mice, as indicated by the increased Cr contents and MDA levels, which was consistent with a previous study using rats exposed to Cr (VI)^27^. Supplementation with *P. acidilactici* BT36 remarkably increased Cr excretion, decreased Cr accumulation in the liver, resulting in lowering hepatic oxidative stress, and alleviating liver damage, which were consistent with the protection of *L. plantarum* on Pb and Cd^8,9^.

BT36 with strong antioxidation mitigated the adverse effects of Cr(VI) by reducing oxidative stress. Although most Cr was excreted in feces with BT36 administration, a high level of Cr still remained in the small intestine (Fig. 1b, c). Nonetheless, the level of the inflammation biomarker TNF-α in the BT36 + Cr(VI) group was normal as in the control (Fig. 2f). Restoration of the concentration of TNF-α by BT36 was likely attributed to the antioxidant capability of BT36, which has been demonstrated in vitro (Supplementary Fig. 2b). In fact, *P. acidilactici* reportedly exerts antioxidant effects by increasing antioxidant enzyme activities, inhibiting lipid–peroxidation reactions, scavenging free radicals, and decreasing DNA lesions in tissues^28,29^. Therefore, antioxidation might be one of the main mechanisms for *P. acidilactici* BT36 to alleviate Cr(VI)-induced oxidative stress in mouse tissues.

Successful Cr detoxification in the gut depends on the antioxidant capability of the introduced strain BT36 alone as well as on the GM that could be regulated by BT36. First, the in vitro study showed that BT36 had no evident Cr-binding or reducing ability, i.e., sequestering Cr by direct binding or reduction is not a major route of remediation for BT36. Further, the colonized number of BT36 accounts for less than 1% of the total GM, in line with a previous study showing limited colonization of probiotics after supplementation for the same time length^30^, implying that direct contribution from BT36 is limited during Cr(VI) detoxification. Nevertheless, the protective effect of BT36 was eminent, especially in the enhancement of Cr(VI)-reducing ability of cultured GM (Fig. 3c), which was similar to bioaugmentation that is by using single strain to modify microbial community for enhancing removal of toxic organic pollutants^31–33^, and consistent with our previous study using an *L. plantarum* strain^6^. All the results indicated the GM as one of the downstream effectors of BT36.

As an oxidizing agent, Cr(VI) exerts a great impact on the host as well as on the structure of GM. Consistent with our previous finding, no significant impact of Cr(VI) was observed on bacterial richness or diversity between fecal samples using 16S rRNA sequencing^6^, which was likely due to insufficient activation energy provided to alter a complex microbial system within a limited Cr(VI) intervention period and dosage. Some HMs, like copper, do lead to decrease diversity of GM, others, e.g., Cd, Pb, and aluminum, do not significantly affect GM diversity in mice treated for 8 or 15 weeks^34–36^, suggesting that long-term toxic metal exposure alters the GM in a metal-specific and time-dependent manner^36^. The sequencing results of 16S rRNA varied in some aspects from that of the metatranscriptome, e.g., F/B. Further, metatransciptome profiles might better reflect the contemporaneous responses of the GM to Cr(VI) exposure. Alterations of GM structure at the genus level were observed to be closely associated with oxidative stress in the gut. The abundances of two genera in the GM, namely *Bacteroides* and no_rank_*Lachnospiraceae*, recently identified as biomarkers for the intestinal redox state, showed the corresponding alterations, reflecting an altered redox state in the gut^37^. Consistent with previous reports^34,36,38^, Cr(VI) increased *Clostridium*, *Helicobacter*, *Bacteroides, Parabacteroides*, *Desulfovibrio*, *Prevotella*, and *Mucispirillum*. Some bacteria such as *Clostridium*^39^ and *Helicobacter pylori*^40^ are pathogenic. *Bacteroides* and *Parabacteroides* can produce antagonistic substances such as bacteriocin in response to exogenous toxins (e.g. Cr(VI)) in the intestine^14,41^. The increase in *Desulfovibrio*, *Prevotella*, and *Mucispirillum* is associated with the occurrence of host inflammation^42–44^, thus, blooming of these microbes might be an indicator of inflammation in response to oxidative stress in the intestine^45–47^. Cr(VI) simultaneously decreases the number of beneficial bacteria, such as *Eubacterium*, and *no_rank_Lachnospiraceae*. *Eubacterium* and *Lachnospiraceae* are butyrate-producing bacteria that can promote intestinal and systemic health^48,49^. BT36 conferred protection on the structural balance of the GM by limiting the populations of *Alistipes, Clostridium, Mucispirillum*, *Prevotella, Bacteroides*, and *Parabacteroides* and by restoring the *Eubacterium* and no_rank_*Lachnospiraceae*. *Alistipes* was reported to be positively correlated with oxidative stress^37^, and a decrease in those after BT36 administration indicated amelioration of oxidative stress in the GM. Besides, members of *Faecalibaculum* were blooming in the BT36 + Cr(VI) group, which may expand lactic acid production according to Lim et al.^50^. *Faecalibaculum*, *Eubacterium*, and *Lachnospiraceae* were some of the dominant active taxa in the gut microbial community of the BT36 + Cr(VI) group, which may be the major contributors to resist Cr(VI)-induced oxidative stress. Similar tendencies of *Faecalibaculum* and *Lachnospiraceae* have been observed in mice co-exposed to benzo[a]pyrene and isoorientin, where isoorientin as a strong antioxidant alleviated the benzo[a]pyrene induced oxidative stress of the colon^51^. The trend changes of *Helicobacter* and *Parabacteroides* in this study were consistent with the intervention of potent antioxidant tea polyphenols in mice receiving a high fat diet^37^. Collectively, modulation of the GM by antioxidant BT36 was associated with maintenance of the intestinal redox state.

The GM naturally harbors genes related to Cr(VI) remediation (Supplementary Data 1), as Claus et al.^52^ reported that GM had an extensive capacity to remediate various environmental chemical toxicities through the expression of relevant enzymes. Oxidative stress is an important aspect of HM toxicity and antioxidant enzymes are considered to be the first line of cellular defense against oxidative damage^25,26^. To find genes and enzymes in GM involved in Cr(VI) remediation, 788 DEGs were obtained by screening, and similar numbers were previously obtained in the metatranscriptome analysis of GM in Cd stress^13^. Under Cr(VI) stress, the expression of some genes relevant to antioxidation in the GM was low or even not expressed (Supplementary Data 1), implying the adaption of GM to external stress^53^. Considering the phenotype in the liver and intestine, it is evident that neither the host nor its GM is competent to cope with Cr(VI) stress with their natural antioxidant defense system alone. The presence of BT36 greatly enhanced the expression of genes relevant to Cr(VI) remediation in the GM (Supplementary Data 1). First, upregulation of SOD, CAT, rubredoxin, rubrerythrin, thiol peroxidase, and peroxiredoxin was observed, which may directly or indirectly scavenge reactive oxygen species (ROS). SOD and CAT are important players in the antioxidant defense system, which increases in Crohn’s disease mice receiving lactic acid bacteria^54^. Rubrerythrin and rubredoxin are alternatives to the SOD-CAT in *Desulfovibrio vulgaris*^55^. The role of rubrerythrin in H_2_O_2_ reduction has been verified in several organisms^56–58^. Thiol peroxidase has been reported to be an H_2_O_2_ receptor and reduces hydroperoxides with thiols^59^. Peroxiredoxin is a family of multifunctional thioredoxin-dependent peroxidases that also has potential in antioxidation defense in bacteria^60^. Upregulated antioxidant genes in the GM may function to diminish the concentration of ROS produced by Cr(VI). On the other hand, upregulated DEGs annotated to thioredoxin, flavodoxin, FAD/NAD(P)-binding oxidoreductase, desulfoferrodoxin, and flavin reductase might also provide protection against Cr(VI) toxicity, probably by transforming Cr(VI) to the less toxic Cr(Ш), and thus immobilizing Cr(VI). Thioredoxin is involved in Cr(VI) reduction in various organisms^22,61^. Flavodoxin including the known Cr(VI)-reductase *Chr*R is actually a family with bound FMN that can reduce Cr(VI)^62^. FAD/NAD(P)-binding oxidoreductase is also a flavoprotein with non-covalently bound FMN, which utilizes NAD(P) as an electron donor, generating Cr(V) as an intermediate in reducing Cr(VI)^63^. Flavin reductase with Cr(VI)-reducing capacity has been reported from *Pseudomonas syringae* and also from *E. coli*^64,65^. Taken together, BT36 increased the antioxidant defense and Cr(VI)-reduction ability of the GM by upregulating the expression of relevant genes.

In addition to DEGs whose function has been annotated, many genes participating in gut remediation of toxic metals are still functionally unknown. In the present study, most of the 10 randomly selected DEGs with unknown function were experimentally demonstrated to be highly tolerant to Cr(VI) in vitro (Fig. 5b). Among these, engineered-strain *E. coli* FcrR was found to reduce 84.37% Cr(VI), which was far higher than the control strain. The structural model of FcrR with an FMN mimics the crystal structure of putative flavoprotein (PDB code 3KLB). FMN is essential for electron transfer of flavoprotein^66^. Hence, we speculate that the protein FcrR might reduce chromate by a mechanism similar to flavoprotein, which needs further validation. Besides, there are still numerous upregulated DGEs (208) annotated to hypothetical proteins, whose functions need to be verified. At least part of these DEGs might also be responsible for Cr(VI) resistance or reduction.

Contamination of the human diet by HM is a persistent issue worldwide. Therefore, effective remediation methods targeting the human body are necessary. Gut remediation is a simple, economic, and effective measure to detoxify the human body of foodborne contaminants by introducing specific probiotics as dietary supplements^6,67^. Other antioxidants, such as chemical drugs and plant extracts, might be partially digested by gastric fluids or metabolized by GM^68^, leading to short-term effectiveness. Probiotics can better survive and colonize in the gastrointestinal tract and may lead to a much longer-lasting effect on bodily health. Humans are more exposed to mixed HMs than single one in real life, and HMs threat human health by inducing oxidative stress. In this study, probiotic BT36 having strong antioxidant capacity successfully mitigated Cr(VI) toxicity, which might also provide a promising strategy for alleviating various HMs toxicity.

## Methods

### Evaluation of Cr(VI)-resistant probiotic strains

Nine *P. acidilactici* strains were generously provided by Professor Xusheng Guo (Lanzhou University, China), which were all isolated from dairy products of the Tibetan plateau in China. The strains were separately incubated in 100 mL of Man, Rogosa, and Sharpe (MRS) broth (Solarbio Sci & Tech, Beijing, China) containing 100–4000 μM Cr(VI) (K_2_Cr_2_O_7_, Shuangshuang Chemical Reagent Company, Yantai, China) on a rotary shaker incubator at 160 rpm for 18 h at 37 °C. After OD_600_ measurement, the supernatant was collected by centrifugation to determine Cr(VI) concentration by the DPC method^69^.

### Preparation of probiotic suspension and chow

*P. acidilactici strains* BT36 and XS40 were selected for the mouse experiment (Supplementary Fig. 2a). Strains were stationary cultured in MRS broth at 37 °C overnight. Cells were then collected and resuspended in autoclaved skimmed milk to reach a CFU (colony-forming units) of 10^9^, which were then separated into 10 mL aliquots and stored at −80 °C until use. Each aliquot was thawed to room temperature before administered to mice by oral gavage. K_2_Cr_2_O_7_ was added to the standard commercial rodent chow (Beijing Keaoxieli Feed Co. Ltd) to a final level of about 300 mg Cr(VI)/kg chow (Supplementary Fig. 6). The dose of Cr(VI) was set based on a preliminary experiment (the mass ratio of water consumption to food intake was ~3 to 1)^6^.

### Mouse experiments

All animal protocols were approved by the Institutional Animal Care and Use Committee of Lanzhou University following the Animal Welfare Act guidelines. In total, 48 four-week-old female Kuming mice were purchased from the Experimental Animal Center of Lanzhou University. Upon arrival, mice were individually housed under standard temperature (20 ± 2 °C) and light-dark cycle (12 h:12 h) conditions in a specific-pathogen-free facility. Before starting the experiments, the mice were fed commercial standard chow for 10 days to acclimate, and were then randomly assigned (8 mice per group) to six groups (Fig. 1a). Mice were given ad libitum access to food and water. Body weight and food intake was recorded every week. During the 30 days of the experimental period, mice were placed in clean cages and feces were collected on day 1, 3, 5, 6, 20, and 30, respectively. After sacrifice, the liver, kidney, and small intestine samples were precisely dissected, flushed, weighed and vacuum frozen-dried (LABCONCO FreeZone®). All tissue samples for biochemical analysis and feces samples were immediately placed in liquid nitrogen and stored at -80 °C until use.

### Quantification of BT36 populations in feces

The DNA extraction procedure was strictly performed following the kit instruction (TIANGEN Biotech (Beijing) Co., Ltd, DP328). Universal primers^70^ were used for total bacterial quantification and *P. acidilactici* -specific primers^71^ were used for BT36 quantification (Supplementary Table 4). The absolute quantification was performed by quantitative PCR, and the relative quantification of BT36 in the feces was expressed as the ratio of the absolute count of *P. acidilactici* to the total bacteria^6^. The Cr(VI) reduction ability of fecal bacteria was determined as our previous study^6^ with minor modifications that using YCFA medium^72^. After that, the supernatant was oxidized from Cr(III) to Cr(VI) using KMnO_4_^73^ to ensure the correctness of bacterial reduction.

### Quantification of Cr in tissues and feces

Tissues and feces were immersed in concentrated nitric acid (m/v = 1:5) overnight and digested in a digestion furnace (Shanghai Ciliometric Instrument Co. Ltd, HYP-320) at 300 °C for clarification. The digested solution was transferred to a 25-mL volumetric flask, diluted with ultrapure water, and filtered. The Cr content was determined by inductively coupled plasma mass spectrometry (Agilent Technologies, 7800 ICP-MS). The final concentration of Cr in the tissues and feces was calculated based on the wet weight of each sample.

### Biochemical analysis of tissues

Frozen tissues were weighed and proportionally (w/v, 1/9) immersed in ice-cold 0.9% (w/v) NaCl, and then disrupted using a hand hold homogenizer. After centrifugated at 3000 × *g* at 4 °C for 10 min, suspension was obtained to determine the levels of malondialdehyde (MDA), glutathione peroxidase (GSH-PX), glutamic oxalacetic transaminase (AST), and catalase (CAT) in the liver and the levels of tumor necrosis factor-α (TNF-α) in the small intestine (Jiancheng Bioengineering Institute, Nanjing, China). The analysis was performed according to the manufacturer’s instructions. MDA can react with thiobarbituric acid to form a colored complex that has a maximum absorbance at 532 nm and results were expressed as nmol/mg protein. GSH-Px activity was determined by measuring the decrease of GSH per min on the base of its catalysis. One unit of the enzyme activity was defined as a decrease of 1 μmol/L GSH per min for 1 mg tissue protein and the results were expressed as U/mg protein. CAT activity was determined by the decrease of the H_2_O_2_ at 240 nm and expressed as U/mg protein. TNF-α were measured using ELISA. Total protein concentration was determined by the Coomassie protein assay.

### Histological analysis

Freshly dissected liver samples were fixed with 4% paraformaldehyde (Shzychemco., Ltd CAS:30525-89-4) overnight and processed by successive dehydration in ethanol and clarified in xylene. Samples were then embedded in paraffin, sectioned at 5-µm thickness using a semi-auto microtome (Leica Biosystems) and stained with hematoxylin and eosin^74^ using an autostainer (Leica Biosystems). The specimens were observed and photographed under a light microscope (Leica Biosystems).

### DNA extraction and processing for sequencing

The fresh feces excreted by mice in sterile cages were immediately collected in 2 mL sterile centrifuge tubes. Total bacterial DNA was isolated from fecal samples following the kit instructions (Tiangen Biotech (Beijing) Co., Ltd, DP328). Extracted DNA was used as a template for PCR amplification of bacterial 137 °C S rRNA genes. PCR conditions, library preparation, and sequencing were performed as described previously^6^. Bacterial diversity was studied by Illumina MiSeq sequencing of the amplified V4-V5 region of 16S rRNA. The resulting sequence read files were carried out by using QIIME 2 pipeline (Quantitative Insights Into Microbial Ecology; http://qiime2.org) and its plugins. Specifically, the ‘demux’ plugin (https://github.com/qiime2/q2-demux) was used for the import of the demultiplexed paired-end sequencing reads and the creation of the ‘artifact’ file (i.e., qiime2 data format required for subsequent analyses). Further, the ‘DADA2′ plugin^75^ was applied for quality filtering (–p-max-ee 2,–p-trunk-q 2), chimera filtering (‘consensus’), to trim primers (–p-trim-left-f 23,–p-trim-left-r 20), to truncate forward and reverse reads (–p-trunc-len-f 200,–p-trunc-len-r 200), and finally to collapse reads into amplicon sequence variants (ASVs). Samples were rarefied to 28,677 reads per sample for subsequent analysis to reduce the bias due to different sequencing depths. Taxonomy to these ASVs was assigned against the Greengenes database (version 13_8) by using the ‘feature-classifier’ plugin (https://github.com/qiime2/q2-feature-classifier) with the ‘fit-classifier-sklearn’. Furthermore, we constructed the phylogenetic tree using muscle and Fast Tree 2^76^.

### RNA extraction and sequencing processing

Fresh feces were collected as above, frozen in liquid nitrogen immediately, and stored at −80 °C until further processing. Feces from three mice in each group were mixed before RNA extraction to reduce individual variation. Total RNA was isolated using RNeasy Mini Kit (Qiagen, Germany). RNA purity and concentration were verified with a Nanodrop spectrophotometer (Agilent Alto, CA, USA). The quality of RNA was checked by 1% agarose gel electrophoresis and using a 2100 Bioanalyzer (Agilent Technologies, Palo Alto, California). RNA samples with RNA integrity number scores higher than 7.0 were used for metatranscriptomic sequencing. Total RNA samples were treated with the Ribo-Zero rRNA Removal kits (Epicentre) to deplete environmental rRNA before subsequent library construction using the TruSeq™ RNA Sample Prep Kit. Bridge PCR was performed before RNA was sequenced on an Illumina Hiseq (HiSeq 3000/4000 SBS Kits) X Ten platform (Majorbio).

To improve the quality and reliability of follow-up analysis, sequencing adaptors were removed from the raw sequencing data, followed by clipping the nucleotides <50 from the reads using Seqprep (http://github.com/jstjohn/SeqPrep). After clipping, the remaining reads shorter than 50 nucleotides, the bases with low-quality (*Q* < 20) and poly-N from the reads were removed using Sickle (http://github.com/najoshi/sickle). *Mus musculus* genome sequences were downloaded from Ensembl Web (http://www.ensembl.org/info/data/ftp/index.html) to remove the host sequences from microbial data sets using BWA (http://bio-bwa.sourceforge.net). RNA sequences were further aligned to the SILVA SSU (16S/18S) and SILVA LSU (23S/28S) database using SortMeRNA software (http://bioinfo.lifl.fr/RNA/sortmerna) to remove any rRNA sequences. All clean data were then employed for de novo assembly using Trinity (http://trinityrnaseq.github.io, version: trinityrnaseq-r2013-02-25), and transcripts of length greater than or equal to 300 bp were selected for subsequent processing. All assembled transcripts were subjected to ORF prediction using TransGeneScan software (http://sourceforge.net/projects/transgenescan). Then, the transcripts of all the four samples were combined and clustered into unique classes with CD-HIT (parameters were set at 95% identity, 90% coverage). After the assembly and clustering of transcripts, the longest sequence of each class was treated as unigene. These unigenes were applied for downstream bioinformatic analysis.

The transcripts per million (TPM) measures were used as an estimation of expression^77,78^. The quality of the assembled sequences was assessed by mapping the quality-filtered paired-end reads to the assembled sequences using Bowtie (version 2.2.3) with the default parameters implemented in RSEM (RNA-Seq by Expectation Maximization). For taxonomic profiling, all unigenes were compared with the NCBI Non-redundant protein (Nr) database using BlastP (BLAST version 2.2.31+, http://blast.ncbi.nlm.nih.gov/Blast.cgi). The parameter *E*-value < 1e^−5^ of BLAST was used to determine statistically significant differences.

### Differential expressed genes (DEGs) analysis

All differential expression analyses were performed using the R package edgeR. The Benjamini-Hochberg method was employed to correct the *P* value of the results of DEG analysis with the false-discovery rate (FDR) for multiple comparisons. The value of log2-fold-change (log_2_FC) ≥ 1, as well as TPM > 100 in at least one of the samples were taken as the selection criteria.

### Plasmid construction and bacterial transformation

The DEGs of interest were commercially synthesized by TSINGKE Biological Technology. DNA was amplified with PrimeSTAR HS DNA Polymerase (TaKaRa). The DEGs were subcloned into different restriction enzymes sites (Supplementary Table 5) before inserting them into the plasmid pET28a. Plasmid constructs were transformed into *E. coli* BL21(DE3) and verified by DNA sequencing (TSINGKE Biological Technology). To test the Cr(VI) resistance of transformed bacterial cells, 11 BL21 strains were grown in 10 mL Luria-Bertani (LB) medium supplemented with 100 μg/ml of kanamycin. After culture to an OD_600_ of 0.6–0.8 at 37 °C, 1 mM Cr(VI) and 500 μM isopropyl-β-D-thiogalactopyranoside were added, and the cultures were then incubated at 16 °C. After 16 h, the cell growth was measured using a spectrophotometer at 600 nm. To determine the Cr(VI)-reduction ability of the strains, cell-free extracts were prepared as described previously^61^. A BCA kit (Solarbio, (Beijing) Co. Ltd.) was used to determine the total protein concentration. The enzyme activities of strains were detected using 5-mL reaction mixtures with 1 mM Cr(VI), along with 3 mM NADH as an electron donor. After 6 h incubation, the concentration of residual Cr(VI) in the supernatant was measured as described above.

### Transmission electron microscopy (TEM) analysis

The engineered strain was inoculated in LB liquid medium with or without 1 mM Cr(VI) and incubated overnight. The cells were then harvested, washed three times with PBS (pH 7.0), and fixed with 2.5% glutaraldehyde (Solarbio, (Beijing) Co. Ltd.). The later steps were performed according to a previously reported method^79^. Ultrathin sections were obtained and then viewed using TEM (Tecnai F30, FEI). Energy dispersive X-ray (EDX) analysis (Tecnai F30, FEI) was performed to detect the elemental composition of the cells.

### Enzyme model of hypothetical protein

The X-ray crystal structure of a putative flavoprotein (PDB code 3KLB) from *Bacteroides fragilis* was downloaded from PDB and was used as a template model. The secondary structure of FcrR was predicted using I-TASSER (https://zhanglab.ccmb.med.umich.edu/I-TASSER/). The protein structure was mapped using PyMOL (http://www.pymol.org/).

### Statistics and reproducibility

Data were expressed as the mean ± SEM. Data significance was analyzed using student’s *t*-test when two groups were compared or using the one-way analysis of variance (ANOVA) followed by Tukey’s post hoc test when more than two groups were compared (Graph Pad Prism 6). Significance was marked as *p < 0.05, **p < 0.01, ***p < 0.001, and ****p < 0.0001. Statistical Analysis of Metagenomic Profiles (STAMP) v2.0.0 software^80^ was employed to test for statistically significant differences (*p* < 0.05) between the fecal microbiome of groups, using ANOVA with Benjamini-Hochberg FDR^81^ multiple test correction or one sided *t*-test. A heat map was constructed using the OmicShare tools, a free online platform for data analysis (http://www.omicshare.com/tools). No data point was excluded in all analyses. Sample size accord with standard practice in the field. All data presented in this study derived from representative independent experiments.

### Reporting summary

Further information on research design is available in the Nature Research Reporting Summary linked to this article.

## Supplementary information

Supplementary Information

Description of Additional Supplementary Files

Supplementary Data 1

Supplementary Data 2

Reporting Summary

Peer Review File

## Data Availability

All the sequences supporting the findings of the study have been deposited in the National Center for Biotechnology Information (NCBI) Sequence Read Archive (SRA). The raw 16S rRNA gene sequence data for the feces microbiota were deposited under number PRJNA545583, the raw metatranscriptome sequence data for the feces microbiota were deposited under number PRJNA547714, and the draft genome sequence of BT36 were deposited under number PRJNA551092. The newly generated plasmids were deposited in the Addgene under number 78023. Source data underlying the graphs presented in the main figures are available in Supplementary Data 2. All other data (if any) are available upon reasonable request.
