# Supplementary material for: Tibet plateau probiotic mitigates chromate toxicity in mice by alleviating oxidative stress in gut microbiota
Source: Commun Biol. 2020 May 15;3:242. doi: 10.1038/s42003-020-0968-3 (PMC7229148; doi:10.1038/s42003-020-0968-3)
Supplement: Supplementary file 1 — Supplementary Information [file 42003_2020_968_MOESM1_ESM.pdf]

Supplementary Information

Supplementary Figures

Supplementary Figure 1. **a** Total chow consumption per mouse per day in the whole experiment. **b** Effect of Cr(VI) and probiotics on growth rate of mice. Values are mean± SEM (n=5 per group).

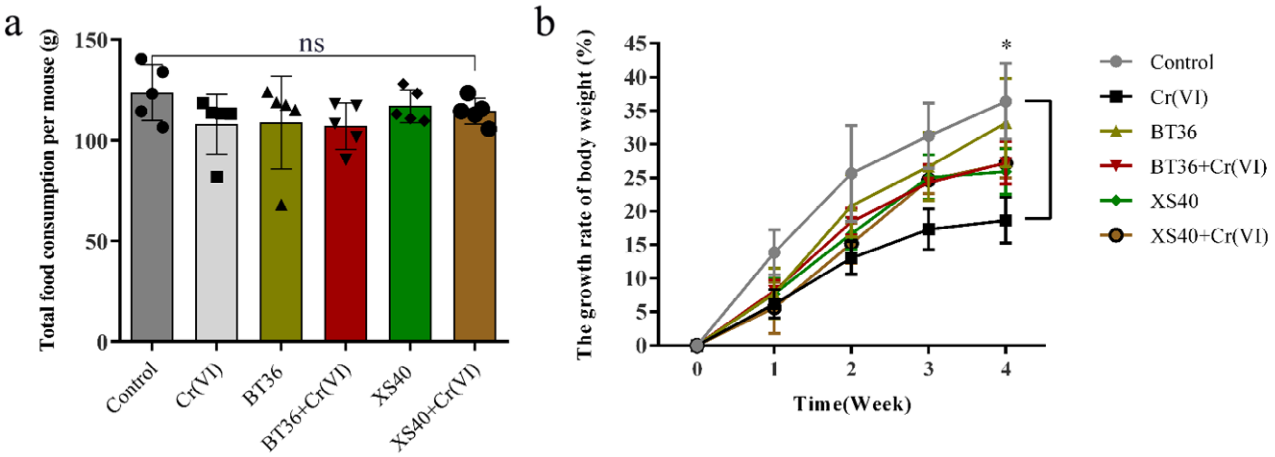

Supplementary Figure 2. a The growth of multiple *Pediococcus acidilactici* at different concentration of hexavalent chromium. **b** The free radical scavenging capacity of BT36 and XS40. • OH, hydroxyl radical; DPPH, 1,1-Diphenyl-2-picrylhydrazyl radical 2,2-Diphenyl-1-(2,4,6-trinitrophenyl) hydrazyl radical.

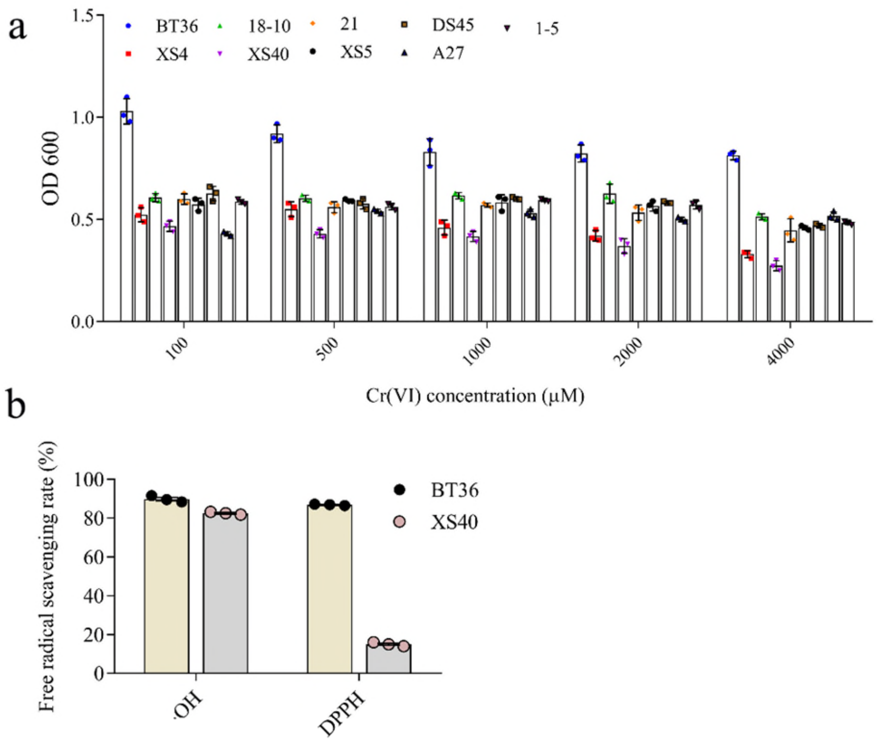

Supplementary Figure 3. The sequence length distribution of four groups.

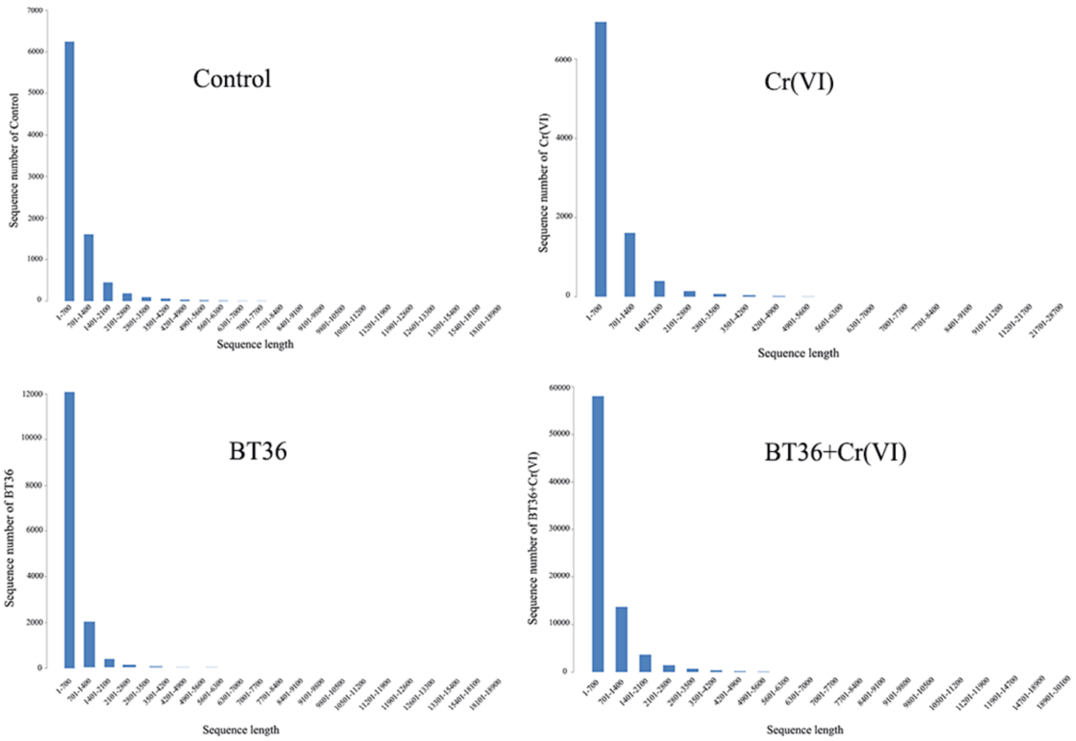

Supplementary Figure 4. The expression of the most important genes related to Cr(VI) reduction and antioxidation.

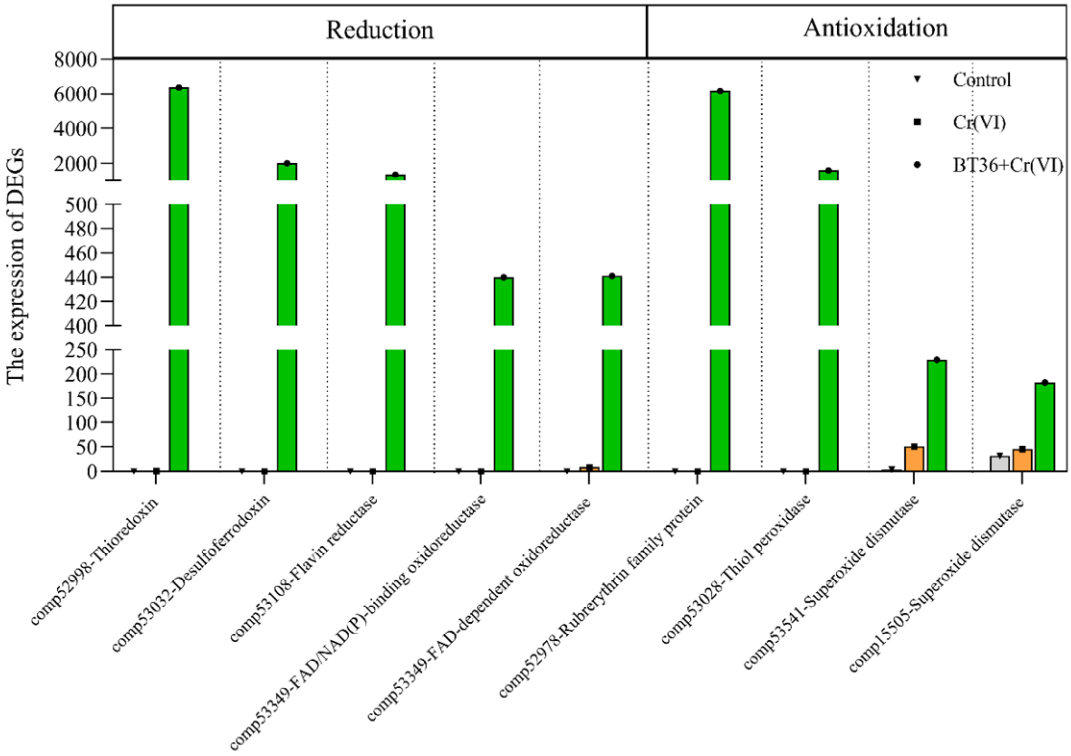

Supplementary Figure 5. qPCR analysis of the expression profiles of DEGs in the gut microbiota of mice (n=3 per group).

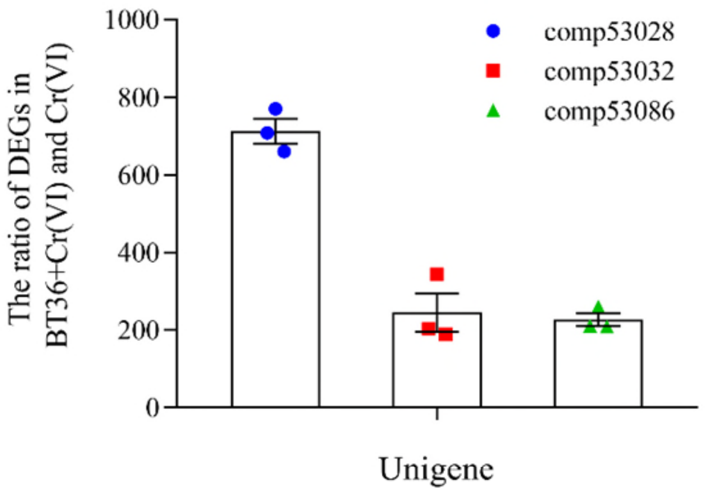

Supplementary Figure 6. The concentration of Cr content in the standard commercial rodent chow and the modified rodent chow with Cr(VI) added. Values are mean± SEM (n=3 per group).

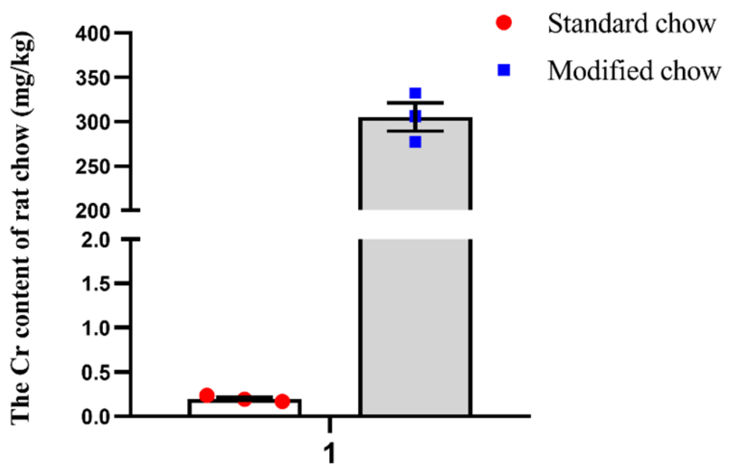

Supplementary Tables

Supplementary Table 1.

Biodiversity measures of total microbiota based on 16S rRNA sequencing.

Sample ID	Shannon	Chao1	Observed	Simpson
Control-1	4.028	335.056	332	0.046
Control-2	4.139	404.000	390	0.052
Control-3	4.398	520.022	499	0.059
BT36-1	3.771	343.070	342	0.109
BT36-2	3.906	319.667	315	0.084
BT36-3	4.011	604.920	558	0.103
Cr(VI)-1	3.929	265.000	265	0.061
Cr(VI)-2	3.592	480.923	452	0.129
Cr(VI)-3	3.762	398.500	388	0.128
BT36+Cr(VI)-1	3.783	273.125	273	0.089
BT36+Cr(VI)-2	3.931	436.484	426	0.103
BT36+Cr(VI)-3	3.783	280.667	279	0.098

Supplementary Table 2.

Statistics of assembly result.

Sample	Seqs	Seqs bases (bp)	N50 (bp)	N90 (bp)	Max (bp)	Min (bp)
BT36	14659	8174450	556	329	9575	300
BT36+Cr(VI)	78611	55770480	813	347	29842	300
Control	87739	67965177	945	358	28959	300
Cr(VI)	92503	61891539	738	344	28282	300

Seqs: The number of sequences in a transcript; Seq bases: The total sequence length of a transcript; N50（N90）: Sequence the transcripts in order of length, scan the length of each sequence one by one from the largest to the smallest, and accumulate. When the accumulated value exceeds 50% (90%) of the total length of all sequences for the first time, the length value of the scanned sequence is N50 (N90); Compared with the average length of the sequence, N50 (N90) can more accurately represent the splicing effect of sequence. Max: The sequence length of the longest transcript; Min: The sequence length of the shortest transcript.

Supplementary Table 3.

Percentage of active bacteria in each sample at the phylum level. It is a default to combine all species whose expression abundance is less than 1% in all samples into others.

**Percentage**	**Control (%)**	**Cr(VI) (%)**	**BT36 (%)**	**BT36+Cr(VI) (%)**
Firmicutes	45.74	35.89	27.37	67.3
Bacteroidetes	46.65	55.8	24.26	23.89
Proteobacteria	4.98	4.37	11.01	4.4
Actinobacteria	0.36	0.55	0.2	2.26
Norank_d_*Eukaryota*	0.04	0.11	29.17	0.2
Norank_d_norank	0.38	1.17	2.37	0.98
Others	1.85	2.10	5.62	0.97

Supplementary Table 4.

Primer used in this study.

**Primer name**	**Sequence (5’- 3’)**	**PCR product size (bp)**	**Ref.**
Total bacteria, RT, F	GCAGGCCTAACACATGCAAGTC	340	[1]
Total bacteria, RT, R	CTGCTGCCTCCCGTAGGAGT		
BT 36, RT, F	GGACTTGATAACGTACCCGC	449	[2]
BT 36, RT, R	GTTCCGTCTTGCATTTGACC		
MiSeq16SV4, 515F	GTGCCAGCMGCCGCGGTAA	394	[3]
MiSeq16SV5, 909R	CCCCGYCAATTCMTTTRAGT		
comp53028, RT, F	AGTCACGGATGTGAATCTGGC	384	This study
comp53028, RT, R	GCCTTGCTGTAATCCGGTTC		
comp53086, RT, F	GTCTGCCTTCTTCGCGCTG	375	This study
comp53086, RT, R	CATTCCGGTACCATTGTGGCA		
comp53032, RT, F	CGGTGCTCTGGTGGAAGTT	279	This study
comp53032, RT, R	AACACAGCCACCGGTTCTTC		
comp53028, RT, F	AGTCACGGATGTGAATCTGGC	384	This study
comp53028, RT, R	GCCTTGCTGTAATCCGGTTC		
comp53086, RT, F	GTCTGCCTTCTTCGCGCTG	375	This study
comp53086, RT, R	CATTCCGGTACCATTGTGGCA		
comp53032, RT, F	CGGTGCTCTGGTGGAAGTT	279	This study
comp53032, RT, R	AACACAGCCACCGGTTCTTC		

Supplementary Table 5.

The list of restriction enzymes sites of the DEGs interest.

Gene name	Gene length (bp)	Restriction enzyme sites
comp53047	396	EcoR I (192)/Hind III (173)
comp53095	258	EcoR I (192)/Hind III (173)
comp24337	279	EcoR I (192)/Hind III (173)
comp52516	261	EcoR I (192)/Hind III (173)
comp53346	480	EcoR I (192)/Hind III (173)
comp53299	375	EcoR I (192)/Hind III (173)
comp53032	396	EcoR I (192)/Hind III (173)
comp53087	279	Nde I (238)/Sal I (179)
comp51796	1104	EcoR I (192)/Sal I (179)
comp53431	507	NdeI (238)/ XhoI (158)

Supplementary Methods

Preparation of cell-free extracts

BT36 was stationary inoculated in MRS at 37°C for overnight, then transferred into 100 mL fresh MRS at 2% ratio at 37°C for 18 h. The fermentation liquid was centrifuged at 8000 r/min for 10 min at 4°C to harvest the intact bacteria. The bacteria were washed 3 times with 0.02 M PBS (pH 7.2) and suspended in PBS at a concentration of 10^{10} CFU mL^{-1}. for the preparation of cell-free extracts, the bacterial suspension was ice bathed followed by ultrasonic disruption at 400 W for 10 s, 10 s intermittently and the whole process lasted 10 minutes. Then the cell debris was removed by centrifugation at 10 000 r/min, for 10 min at 4°C, and the resulting supernatant kept as the cell-free extract.

Determination of scavenging ability of hydroxyl radical (. OH)

The free radical scavenging capacity of BT36 was conducted according to the method reported [4]. 0.1 milliliter of cell-free extract, 0.4 ml of 0.02 M PBS, 0.1 mL of 1 mM EDTA, 0.1 mL of 10 mM H_2O_2, 0.1 mL of 2 mM ascorbic acid, 0.1mL of 60 mM 2-deoxy-D-ribose and 0.1 mL of 1mM $FeCl_3$ were mixed. After incubation at 37°C water bath for 1 h, 0.1 mL of 20% (m/v) TCA and 1mL of 1.0% TBA were

added. After incubation at 100°C water bath for 15 minutes, the whole mixture was cooled down and measured at 365 nm. 1 mL cell-free extracts were replaced with the same volume of PBS and after the same incubation mentioned above, the data were expressed as A0. For the control sample, 1.1 mL PBS was added instated of adding 1mL cell-free extracts and 0.1 mL deoxyribose, and the data were expressed as AC. Each group was set up with three repeats. The scavenging ability was defined as follows:

$$\bullet \text{OH scavenging rate (\%)} = 1 - \frac{AS-AC}{A0} \times 100\%$$

A0: absorbance of reagent blank reaction without sample;

AS: absorbance of reaction group containing sample and deoxyribose;

AC: absorbance of blank reaction group without deoxyribose.

Determination of scavenging ability of 1,1-diphenyl-2-picrylhydrazyl (DPPH)

The DPPH scavenging abilities were determined as previously described [5]. A mixture of 1.9 mL freshly prepared DPPH solution (0.12 mM) and 0.1 mL cell-free extracts were incubated for 30 min in the dark. The scavenged DPPH was analyzed by measuring the decrease in absorbance at 517 nm. Three repeats were set up for each group. The scavenging ability was defined as follows:

$$\text{DPPH scavenging effect (\%)} = 1 - \frac{A-B}{A0} \times 100\%$$

A0: absorbance of DPPH (1.9 mL DPPH+0.1 mL 50% ethanol) without sample addition

A: absorbance of the sample after reaction with DPPH (1. 9 mL DPPH + 0. 1 mL sample)

B: absorbance of blank sample (1.9 mL 50% Ethanol + 0.1 mL Sample)

Verification of DEGs by qRT-PCR.

Total RNA was extracted from feces as described above. The concentration of total RNA samples was measured using NanoDrop 2000c Spectrophotometer from Thermo Scientific. The first strand cDNA was transcribed using PrimeScriptTM RT reagent

kit with gDNA Eraser (TaKaRa, Japan), following the manufacturer protocol. PCR was done using a Real Time PCR System (Bio-rad, American) and SYBR Premix EX TaqTM (Tli RNaseH Plus), Bulk (TaKaRa, Japan). After cDNA synthesis, the concentrations of the first-strand samples were brought to 20 ng/ml, which were used as the template in qRT-PCR reactions. Cycling parameters included 95 ^{o}C for 30s, 95 ^{o}C for 5s and then, 60 ^{o}C for 30s, 50 cycles of 95 ^{o}C for 5 s and 60 ^{o}C for 30 s, 95 ^{o}C 10s, 65-95 ^{o}C 5s. Quantification of DEGs gene expression in fecal microbiome was based on the Relative Standard Curve method using the threshold cycle (Ct) values. The expression levels of mRNAs were normalized to 16S rRNA and calculated using the 2-ΔΔCt method. All Primers were designed using NCBI (**Supplementary Table 4**).

Supplementary References

1 Castillo, M. *et al.* Quantification of total bacteria, enterobacteria and lactobacilli populations in pig digesta by real-time PCR. *Veterinary Microbiology* **114**, 165-170, doi:https://doi.org/10.1016/j.vetmic.2005.11.055 (2006).

2 Mora, D., Fortina, M. G., Parini, C. & Manachini, P. L. Identification of Pediococcus acidilactici and Pediococcus pentosaceus based on 16S rRNA and ldhD gene-targeted multiplex PCR analysis. *FEMS microbiology letters* **151**, 231-236, doi:10.1111/j.1574-6968.1997.tb12575.x (1997).

3 Tamaki, H. *et al.* Analysis of 16S rRNA Amplicon Sequencing Options on the Roche/454 Next-Generation Titanium Sequencing Platform. *PLOS ONE* **6**, e25263, doi:10.1371/journal.pone.0025263 (2011).

4 Halliwell, B., Gutteridge, J. M. C. & Aruoma, O. I. The deoxyribose method: A simple "test-tube" assay for determination of rate constants for reactions of hydroxyl radicals. *Analytical Biochemistry* **165**, 215-219, doi:https://doi.org/10.1016/0003-2697(87)90222-3 (1987).

5 Chen, P. *et al.* Screening for potential new probiotic based on probiotic properties and α-glucosidase inhibitory activity. *Food Control* **35**, 65-72, doi:https://doi.org/10.1016/j.foodcont.2013.06.027 (2014).
