# Supplementary material for: Tibet plateau probiotic mitigates chromate toxicity in mice by alleviating oxidative stress in gut microbiota
Source: Commun Biol. 2020 May 15;3:242. doi: 10.1038/s42003-020-0968-3 (PMC7229148; doi:10.1038/s42003-020-0968-3)
Supplement: Supplementary file 2 — Description of Additional Supplementary Files [file 42003_2020_968_MOESM2_ESM.pdf]

Description of additional supplementary items

Supplementary Data 1

Relative expression of genes related to Cr(VI) reduction and antioxidation that were unchanged or upregulated in Cr(VI) and displayed a large fold change in the BT36 plus Cr(VI) sample. Only genes expressing $\log_2$ FC ≥ 1.0 were considered.

Supplementary Data 2

Source data underlying the graphs in figures and supplementary Information as an excel file.
