# Supplementary material for: Tibet plateau probiotic mitigates chromate toxicity in mice by alleviating oxidative stress in gut microbiota
Source: Commun Biol. 2020 May 15;3:242. doi: 10.1038/s42003-020-0968-3 (PMC7229148; doi:10.1038/s42003-020-0968-3)
Supplement: Supplementary file 3 — Supplementary Data 1 [file 42003_2020_968_MOESM3_ESM.pdf]

Gene ID	Samples (TPM)			Fold change ir	P-value	FDR
	BT36+Cr(VI)	Control	Cr(VI)			
**Reduction**						
comp52998_c0_seq	6357.047	0	0.9497	12.7086	8.34E-28	2.71E-24
comp53032_c0_seq	2012.036	0	0	10.9744	2.00E-24	2.29E-21
comp53086_c0_seq	1439.4555	0	0.2006	12.8091	1.49E-31	3.47E-27
comp15415_c0_seq	1388.7344	0.1859	4.6896	8.2101	6.46E-22	3.92E-19
comp53108_c0_seq	1361.5582	0	0.435	11.6121	1.40E-24	1.64E-21
comp53172_c0_seq	820.2077	0	0	9.6798	9.33E-22	5.45E-19
comp53183_c0_seq	593.2969	0	1.0101	9.2126	2.46E-22	1.69E-19
comp53349_c0_seq	440.417	0	0.1675	11.3601	8.85E-24	8.63E-21
comp53349_c0_seq	441.3075	0	0	8.7856	4.08E-24	4.28E-21
comp53221_c0_seq	413.5606	36.138	83.1198	2.3148	3.73E-05	5.25E-04
comp53404_c0_seq	388.5555	0	0	8.602	2.19E-22	1.55E-19
comp53492_c0_seq	350.9119	0	0	8.455	7.63E-23	6.12E-20
comp55818_c0_seq	294.7384	0	0	8.2033	2.53E-16	3.88E-14
comp53616_c0_seq	241.6433	0	0	7.9167	1.45E-17	2.94E-15
comp53541_c0_seq	230.1523	3.8874	51.4973	2.16	6.51E-05	8.53E-04
comp67228_c0_seq	189.2899	4.0733	36.2718	2.3837	4.34E-05	6.06E-04
comp53832_c0_seq	157.7314	1.5786	32.7598	2.2675	4.89E-05	6.65E-04
comp53911_c0_seq	138.4604	0	0	7.1133	1.66E-17	3.33E-15
comp15333_c0_seq	136.9528	0	0	7.0975	4.61E-21	2.22E-18
comp54344_c0_seq	125.2589	15.8389	61.2375	1.0324	9.29E-13	7.13E-11
**Antioxidation**						
comp52978_c0_seq	6170.3658	0.4838	0.5083	13.5675	1.97E-30	1.83E-26
comp53028_c0_seq	1593.492	0	0	10.638	4.82E-25	6.23E-22
comp65675_c0_seq	377.6587	1.3331	58.7155	2.6853	3.40E-06	6.49E-05
comp12431_c0_seq	296.261644	2.2786	78.0226	1.9249	2.11E-04	2.36E-03
comp53635_c0_seq	249.7676	5.8681	28.7589	3.1185	3.76E-07	9.09E-06
comp12870_c0_seq	246.5176	0.9605	1.0165	7.9219	9.09E-16	1.22E-13
comp53711_c0_seq	196.1017	69.8265	77.0592	1.3476	3.31E-03	2.25E-02
comp53822_c0_seq	199.0934	3.7531	75.6673	1.3957	2.61E-03	1.90E-02
comp10887_c0_seq	121.2359	28.7508	57.3308	1.0804	9.30E-03	4.97E-02
comp53541_c0_seq	230.1523	3.8874	51.4974	2.16	6.51E-05	8.53E-04
comp15505_c0_seq	183.3457	31.7524	45.7509	2.0027	1.55E-04	1.82E-03
comp53861_c0_seq	144.6550877	2.8594	57.7457	1.3248	3.38E-03	2.30E-02
comp51497_c0_seq	112.1898	22.6301	38.5	1.543	1.53E-03	1.21E-02
comp10887_c0_seq	121.2359	28.7508	57.3308	1.0804	1.28E-16	2.09E-14
**Unknown function**						
comp53346_c0_seq	525.5945	0	0	9.0378	3.71E-20	1.40E-17
comp53095_c0_seq	1263.5387	0	0	10.3033	3.06E-20	1.18E-17
comp24337_c0_seq	909.9861	0	0	9.83	5.58E-19	1.55E-16
comp53087_c0_seq	1160.4648	0	0	10.1805	2.20E-20	8.91E-18
comp53032_c0_seq	2012.036	0	0	10.9744	2.00E-24	2.29E-21
comp53431_c0_seq	336.5851	0	0	8.3948	1.91E-18	4.62E-16
comp53299_c0_seq	407.1865	0	0.8095	9.7308	1.16E-16	1.92E-14
comp52516_c2_seq	849.6734	0	0	8.9744	1.14E-24	1.39E-21
comp53047_c0_seq	1353.387	0	1.5119	9.806	2.36E-21	1.23E-18
comp51796_c0_seq	179.5021	0.812	4.2684	5.3942	4.14E-11	2.31E-09

Species information

Thioredoxin [Faecalibaculum rodentium]
Desulfoferrodoxin [Faecalibaculum rodentium]
Pyruvate:ferredoxin (flavodoxin) oxidoreductase [Faecalibaculum rodentium]
Pyruvate:ferredoxin (flavodoxin) oxidoreductase [Eubacterium plexicaudatum]
Flavin reductase [Faecalibaculum rodentium]
Thioredoxin [Faecalibaculum rodentium]
FprA family A-type flavoprotein [Eubacterium plexicaudatum]
FAD/NAD(P)-binding oxidoreductase [Faecalibaculum rodentium]
FAD-dependent oxidoreductase [Faecalibaculum rodentium]
Thioredoxin [Muribaculaceae bacterium DSM 103720]
Thioredoxin-disulfide reductase [Faecalibaculum rodentium]
FprA family A-type flavoprotein [Faecalibaculum rodentium]
Desulfoferrodoxin [Eubacterium plexicaudatum]
NAD(P)H-dependent oxidoreductase [Faecalibaculum rodentium]
superoxide dismutase [Muribaculum intestinale]
Thioredoxin [Muribaculum intestinale]
MULTISPECIES: flavodoxin [unclassified muribaculaceae]
Nitroreductase family protein [Eubacterium plexicaudatum]
Nitroreductase family protein [Faecalibaculum rodentium]
Thioredoxin [Duncaniella muris]

Rubrerythrin family protein [Faecalibaculum rodentium]
Thiol peroxidase [Faecalibaculum rodentium]
Rubredoxin [Muribaculum intestinale]
Peroxiredoxin [Muribaculum intestinale]
Rubrerythrin family protein [Muribaculum intestinale]
NADH peroxidase [Lachnospiraceae bacterium A2]
Peroxiredoxin [Muribaculaceae bacterium DSM 103720]
Rubrerythrin [Muribaculum intestinale]
Rubrerythrin family protein [Parabacteroides distasonis]
Superoxide dismutase [Muribaculum intestinale]
Superoxide dismutase [Duncaniella muris]
MULTISPECIES: manganese catalase family protein [Bacteroidales]
MULTISPECIES: Rubrerythrin family protein [Enterorhabdus]
Rubrerythrin family protein [Parabacteroides distasonis]

hypothetical protein [Faecalibaculum rodentium]
hypothetical protein AALO17_17630 [Faecalibaculum rodentium]
hypothetical protein [Faecalibaculum rodentium]
hypothetical protein [Faecalibaculum rodentium]
hypothetical protein BVX94_00395 [bacterium B17]
hypothetical protein BO223_09690 [Faecalibaculum rodentium]
hypothetical protein [Faecalibaculum rodentium]
hypothetical protein [Faecalibaculum rodentium]
hypothetical protein [Eubacterium plexicaudatum]
Hypothetical protein [Lachnospiraceae bacterium 3-1]
