# Supplementary material for: Tibet plateau probiotic mitigates chromate toxicity in mice by alleviating oxidative stress in gut microbiota
Source: Commun Biol. 2020 May 15;3:242. doi: 10.1038/s42003-020-0968-3 (PMC7229148; doi:10.1038/s42003-020-0968-3)
Supplement: Supplementary file 5 — Reporting Summary [file 42003_2020_968_MOESM5_ESM.pdf]

nature research

Corresponding author(s): Xiangkai Li

Last updated by author(s): Apr 8, 2020

Reporting Summary

Nature Research wishes to improve the reproducibility of the work that we publish. This form provides structure for consistency and transparency in reporting. For further information on Nature Research policies, see Authors & Referees and the Editorial Policy Checklist.

Statistics

For all statistical analyses, confirm that the following items are present in the figure legend, table legend, main text, or Methods section.

n/a	Confirmed	
☐	☒	The exact sample size (*n*) for each experimental group/condition, given as a discrete number and unit of measurement
☐	☒	A statement on whether measurements were taken from distinct samples or whether the same sample was measured repeatedly
☐	☒	The statistical test(s) used AND whether they are one- or two-sided<br>*Only common tests should be described solely by name; describe more complex techniques in the Methods section.*
☒	☐	A description of all covariates tested
☐	☒	A description of any assumptions or corrections, such as tests of normality and adjustment for multiple comparisons
☐	☒	A full description of the statistical parameters including central tendency (e.g. means) or other basic estimates (e.g. regression coefficient) AND variation (e.g. standard deviation) or associated estimates of uncertainty (e.g. confidence intervals)
☒	☐	For null hypothesis testing, the test statistic (e.g. *F*, *t*, *r*) with confidence intervals, effect sizes, degrees of freedom and *P* value noted<br>*Give P values as exact values whenever suitable.*
☒	☐	For Bayesian analysis, information on the choice of priors and Markov chain Monte Carlo settings
☒	☐	For hierarchical and complex designs, identification of the appropriate level for tests and full reporting of outcomes
☒	☐	Estimates of effect sizes (e.g. Cohen's *d*, Pearson's *r*), indicating how they were calculated

Our web collection on statistics for biologists contains articles on many of the points above.

Software and code

Policy information about availability of computer code

Data collection

1) The detection of bacterial density and Cr(VI) concentration was measured by VIS spectrophotometer (V5100, Shanghai).
2) Data of malondialdehyde, glutathione, peroxidase, glutamic oxalacetic transaminase, catalase and tumor necrosis factor-α were measured by microplate reader (Vaarioskan Flash Multimode Reader, Thermo Scientific).
3) The Cr content was determined by inductively coupled plasma mass spectrometry (Agilent Technologies, 7800 ICP-MS).
4) The real-time quantification of BT36 in the feces was measured by Bio-RAD CFX96 (BIO-LAB).
5) Gut microbiota were analyzed by Genesky Biotechnologies Inc., Shanghai using primers that target to the V4-V5 regions of 16S rRNA.
6) The quality of isolated DNA and RNA samples were evaluated with and Agilent Bioanalyzer 2100 (Agilent technologies) and the purified RNA was quantified using a NanoDrop spectrophotometer (Agilent Alto, CA, USA).
7) The metatranscriptome of gut microbiota was analyzed by Majorbio Biotechnology company (Shanghai, China) on an Illumina Hiseq (HiSeq 3000/4000 SBS Kits) X Ten platform.
8) TEM and EDX were measure by transmission electron microscopy (Tecnai F30, FEI).
9) Hematoxylin/eosin staining was analyzed by Leica Biosystems.
10) The secondary structure of FcrR was predicted using I-TASSER (https://zhanglab.ccmb.med.umich.edu/I-TASSER/). The protein structure was mapped using PyMOL (http://www.pymol.org/).

Data analysis	1) Graph Pad Prism 6 was used to analyze data significance using student's t-test when two groups were compared or using the one-way nonparametric Tukey's post hoc test when more than two groups were compared.<br>2) Statistical Analysis of Metagenomic Profiles (STAMP) v2.0.0 software was employed to analyze statistical significance between the fecal microbiome of four groups using ANOVA with Benjamini-Hochberg FDR multiple test correction, and to analyze statistical significance between the fecal microbiome of two groups using one sided t-test.<br>3) Amplicon sequence variants were inferred with DADA2 (v1.6.0). Furthermore, the phylogenetic tree was constructed using muscle and Fast Tree 2.<br>4) OmicShare tools, a free online platform for data analysis (http://www.omicshare.com/tools) was used to generate the heatmap in this study.

For manuscripts utilizing custom algorithms or software that are central to the research but not yet described in published literature, software must be made available to editors/reviewers. We strongly encourage code deposition in a community repository (e.g. GitHub). See the Nature Research guidelines for submitting code & software for further information.

Data

Policy information about availability of data

All manuscripts must include a data availability statement. This statement should provide the following information, where applicable:
- Accession codes, unique identifiers, or web links for publicly available datasets
- A list of figures that have associated raw data
- A description of any restrictions on data availability

1) Raw 16S rRNA gene sequence data for the feces microbiota were deposited in the NCBI Sequence Read Archive under BioProject Accession Number PRJNA545583, which is related to Figure 4A-4D;
2) Raw metatranscriptome sequence data for the feces microbiota were deposited in the NCBI Sequence Read Archive under BioProject Accession Number PRJNA547714, which is related to Figure 4E and Figure 5A;
3) The draft genome sequence of BT36 were deposited in the NCBI Sequence Read Archive under BioProject Accession Number PRJNA551092, which is related to Table1
4) The newly generated plasmids in this study were deposited in the Addgene under number 78023.
5) All other data is available from the Supplementary data 2, which is related to all figures except figures 4 and 5 A,D,E, F,G.

Field-specific reporting

Please select the one below that is the best fit for your research. If you are not sure, read the appropriate sections before making your selection.

☒ Life sciences ☐ Behavioural & social sciences ☐ Ecological, evolutionary & environmental sciences

For a reference copy of the document with all sections, see nature.com/documents/nr-reporting-summary-flat.pdf

Life sciences study design

All studies must disclose on these points even when the disclosure is negative.

Sample size	1) For total chow consumption per mouse per day and growth rate of mice, n=5;<br>2) For Cr levels in feces and tissues of mice, n=5;<br>3) For biochemical analysis of tissues, n=8;<br>4) For quantification of BT36 in feces, n=5;<br>5) For Cr(VI) reduction ability of fecal bacteria, n=3;<br>6) For 16S rRNA analysis, n=3
Data exclusions	No data exclusions
Replication	Except for metatranscriptome sequencing, other experiments were repeated at least three times.
Randomization	Mice were randomly assigned to six groups in this study (8 mice per group).
Blinding	Not relevant for this study.

Reporting for specific materials, systems and methods

We require information from authors about some types of materials, experimental systems and methods used in many studies. Here, indicate whether each material, system or method listed is relevant to your study. If you are not sure if a list item applies to your research, read the appropriate section before selecting a response.

Materials & experimental systems

n/a	Involved in the study
☒	☐ Antibodies
☒	☐ Eukaryotic cell lines
☒	☐ Palaeontology
☐	☒ Animals and other organisms
☒	☐ Human research participants
☒	☐ Clinical data

Methods

n/a	Involved in the study
☒	☐ ChIP-seq
☒	☐ Flow cytometry
☒	☐ MRI-based neuroimaging

Animals and other organisms

Policy information about studies involving animals; ARRIVE guidelines recommended for reporting animal research

Laboratory animals	Female Kunming mice.
Wild animals	No wild animals used in this study.
Field-collected samples	Mice were bred and maintained under specific pathogen-free （SPF） conditions in the Animal Center of Lanzhou University
Ethics oversight	All procedures were conducted according to the Institutional Animal Care and Use Committee of the Model Animal Research Center. Animal experiments were approved by the Institute’s Animal Ethics Committee of Lanzhou University.

Note that full information on the approval of the study protocol must also be provided in the manuscript.
