# Supplementary material for: Tibet plateau probiotic mitigates chromate toxicity in mice by alleviating oxidative stress in gut microbiota
Source: Commun Biol. 2020 May 15;3:242. doi: 10.1038/s42003-020-0968-3 (PMC7229148; doi:10.1038/s42003-020-0968-3)
Supplement: Supplementary file 6 — Peer Review File [file 42003_2020_968_MOESM6_ESM.pdf]

Reviewers' comments:

Reviewer #1 (Remarks to the Author):

This manuscript finds that BT36, one of probiotics, can alleviate chromate toxicity by reducing oxidative stress. It provides solid data to support their hypothesis and investigate the underlying mechanism at the same time. It is a high-quality manuscript, but some questions need to be clarified before considering for acceptance. My concerns are listed below:
1. P2L29: the authors declare that the detoxification is still unclear. In my opinion, there are already lots of papers focusing on this field, some mechanisms have been investigated. It just is not totally clear.
2. P4L101-104 this sentence is too long to be understood clearly.
3. P9L196-198 the authors declare that the diversity of the total bacterial community was stable among different groups. As we know, heavy metals are toxic to gut microbiota, lots of researches find that the diversity of gut microbiota decrease under the toxicity of HMs. Authors need to explain the reason in the discussion.
4. P17L331 restoring the concentration of TNF-α?
5. P18L356 what is F/B?
6. P18L369 I think microbes is better than populations.
7. P20L400-403 some genes of microbes are inducible. They are low or no expressed under stress free environment. But here opposite tendency is found. More explanations are needed.
8. P23L490 what is the purpose of vacuum-dried for tissue samples? Frozen-dry is more used.
9. P23L507 how feces is treated?
10. P24L531 replace microbial by bacterial
11. P24L541 delete an
12. P25L560 change unique unigenes to unigenes
13. P26L597 pH7.0

Reviewer #2 (Remarks to the Author):

Comments to the authors.

This is a very interesting study which could be very relevant for the bioremediation of Cr(VI). The manuscript is well written and the conclusion of the authors corroborated by their results. My major remarks is that more details and a more recent approach could be undertaken for the bioinformatics analysis. In addition, some parts of the presentation of the results lack clarity.

Detailed suggestions.

Line 474. The authors indicate that 'K2Cr2O7 was added to the standard commercial rodent chow (Beijing Keaoxieli Feed Co. Ltd) to a final level of about 300 mg Cr(VI)/kg chow.' Could you indicate if this compound can be found as a background contaminant in the rat feed. Rodent diets are frequently contaminated by heavy metals. Has it been measured?

Results. The clarity of figures should be improved. I think that the authors should consider using another data visualisation strategy. It is very misleading to present plots where the spread of the data cannot be assessed, especially for experiments performed with a low number of replicates. The authors could use a dot plot representation which has become the standard to represent effect sizes in biology (https://journals.plos.org/plosbiology/article?id=10.1371/journal.pbio.1002128).

I know by experience that the intragroup variation can be quite large in this type of gut microbiome experimental design. Could you provide dot plots to assess the effect size and the experimental variations for the most important genes having their expression altered in your study.

The composition of the gut microbiome samples has been studied at the family and genus levels. Tools are available to perform the analysis at the species levels. It appears that the authors have used the QIIME Pipeline v. 1.7.0 which appears to be outdated. I would recommend to use a more recent method including the determination of amplicon sequence variants which have been recommended to replace OTUs and are used in more recent version of QIIME2.

It is always very convenient to have estimations of the number of reads and the quality of a gut microbiome study. Could you indicate how many reads per sample were sequenced and provide more details on the quality of this dataset (both for the metatranscriptomics and the 16S analysis). In general, the bioinformatics pipeline is poorly described and more details could be useful.

Line 519. 'According to the manufacturer's instructions" Could you explain briefly what was the different methods

Line 572. There is a typo; 'flod-change'

It is not clear to me if the authors have used faecal samples collected in the cage or directly from the animals during autopsy. If this is the latter, could you explain which sections of the gastrointestinal tract were sampled. The microbiome can be very different depending on the location of the sampling.

Could you provide indications on the qualities of your transcript assemblies?

I suggest to cite the most recent relevant references on the topic which have been performed in laboratory animals such as https://www.ncbi.nlm.nih.gov/pubmed/31377140

Responses to the Reviewers' Comments

Manuscript Number: COMMSBIO-19-1538

Title: Tibet plateau probiotic mitigates chromate toxicity in mice by alleviating oxidative stress in gut microbiota

Dear editors and reviewers:

We greatly appreciate the reviewers for the insightful comments and suggestions that are very helpful for revising and improving our manuscript. We have read the comments carefully and made corrections accordingly. All comments and answers are listed below.

We hope that this revision will comply with the request. Please do not hesitate to contact us if you need further information.

Pengya Feng

Reviewer #1:

This manuscript finds that BT36, one of probiotics, can alleviate chromate toxicity by reducing oxidative stress. It provides solid data to support their hypothesis and investigate the underlying mechanism at the same time. It is a high-quality manuscript, but some questions need to be clarified before considering for acceptance.

Response: Thank you for the recommendation. We have revised our manuscript according to the detailed comments and the revised parts were highlighted in red.

1. P2L29: the authors declare that the detoxification is still unclear. In my opinion, there are already lots of papers focusing on this field, some mechanisms have been investigated. It just is not totally clear.

Response: We agree with you. Many studies have shown the effects of probiotics on heavy metals detoxification, including intestinal sequestration and intestinal barrier

protection [1-3]. It is just the interaction between probiotics and gut microbiota that is still unclear. Hence we have corrected the sentence on **page 2 line 29-30:**

"Probiotics can protect animals and human against heavy metals, but the detoxification mechanism **has not been fully clarified**."

2. P4L101-104 this sentence is too long to be understood clearly.

Response: Thanks for your reminder. We have rewritten this sentence. Please see **page 4 lines 101-104:**

"In a previous study, an *L. plantarum* strain with strong Cr(VI)-reducing ability effectively diminished Cr accumulation in mouse tissues and enhanced the Cr(VI)-reducing ability of fecal microbes."

3. P9L196-198 the authors declare that the diversity of the total bacterial community was stable among different groups. As we know, heavy metals are toxic to gut microbiota, lots of researches find that the diversity of gut microbiota decrease under the toxicity of HMs. Authors need to explain the reason in the discussion.

Response: Thanks for your comment. In this study, the "stable" means that there was a decrease but no significant changes in the diversity of the total bacterial community in Cr(VI) group compared with the control group, which was **likely due to insufficient activation energy provided to alter a complex microbial system within a limited Cr(VI) intervention period and dosage**. Though some HMs, like copper and arsenic, do lead to decrease diversity of gut microbiota [4,5], others (e.g. cadmium, lead, aluminum, chromium) do not significantly affect gut microbial diversity in mice treated for 8 or 15 weeks [4-7], suggesting that **long-term toxic metal exposure altered the gut microbiota of mice in a metal-specific and time-dependent manner** [4,5]. The relevant discussion has been added on **page 20 lines 372-376** in the revised manuscript.

4. P17L331 restoring the concentration of TNF-α?

Response: Thanks. "Restoration of the concentration" was added on **page 19 lines**

347-348.

5. P18L356 what is F/B?

Response: In this study, F/B is the abbreviation of the ratio of *Firmicutes* to *Bacteroidetes*. We have added full names in the sentence (**page 10 line 203)**.

6. P18L369 I think microbes is better than populations.

Response: We agree. We have replaced "populations" by "microbes" (Please see **page 21 line 391**).

7. P20L400-403 some genes of microbes are inducible. They are low or no expressed under stress free environment. But here opposite tendency is found. More explanations are needed.

Response: Thanks for the comment. We agree with you that some genes were indeed induced in response to Cr(VI) exposure, e.g. in Table 1 the expression of some of the 34 antioxidative genes were moderately upregulated in Cr(VI) v.s. control, suggesting the tendency in this manuscript is in consistence with common knowledge. It is just that the induction or upregulated expression of these genes were not competent enough to defend against Cr(VI) stress, based on the phenotypic results of Cr(VI) group. To avoid misconception, we have deleted 'repression' and made clarification in the discussion part (**Page 22 Line 421 and 425**).

8. P23L490 what is the purpose of vacuum-dried for tissue samples? Frozen-dry is more used.

Response: Thanks for your comment. In here, "vacuum-dried" was meant to be "vacuum frozen-dried". The purpose of using vacuum frozen-dried tissue samples was to thoroughly digest tissues samples with concentrated nitric acid. We have revised the related part in "Method". Please see **page 25 lines 509-510.**

9. P23L507 how feces is treated?

Response: Feces were treated in the same way as tissues [3], and we have added "feces" in "Quantification of Cr in tissues and feces" of Method. Please see

page 25 line 526.

10. P24L531 replace microbial by bacterial

Response: Thanks for your reminder. We have replaced "microbial" by "bacterial" (**page 27 line 562**).

11. P24L541 delete an

Response: Thanks. "an" was deleted on **page 27 line 587**.

12. P25L560 change unique unigenes to unigenes

Response: Thanks. "unique unigenes" was revised as "unigenes" (**page 28 line 615**).

13. P26L597 pH7.0

Response: Thanks for your reminder. We have added "pH" on **P30 L654**.

Reviewer #2:

This is a very interesting study which could be very relevant for the bioremediation of Cr(VI). The manuscript is well written and the conclusion of the authors corroborated by their results. My major remarks is that more details and a more recent approach could be undertaken for the bioinformatics analysis. In addition, some parts of the presentation of the results lack clarity.

Response: Thanks for your comment. We have revised the manuscript according to the detailed comments and highlighted the revised part in red.

(1) Line 474. The authors indicate that 'K2Cr2O7 was added to the standard commercial rodent chow (Beijing Keaoxieli Feed Co. Ltd) to a final level of about 300 mg Cr(VI)/kg chow.' Could you indicate if this compound can be found as a background contaminant in the rat feed. Rodent diets are frequently contaminated by heavy metals. Has it been measured?

Response: Thanks for your comment. We have measured the Cr concentration

of commercial rodent chow before starting animal experiment and we added the figure (**Supplementary Fig. 2**) in Supplementary Information. The results showed that the Cr concentration in the standard rodent chow is about 0.198±0.030 mg/kg, which was lower than China national limit of Cr in foods at 1.0 mg/kg (GB 2762–2017) [8].

Supplementary figure is as follows:

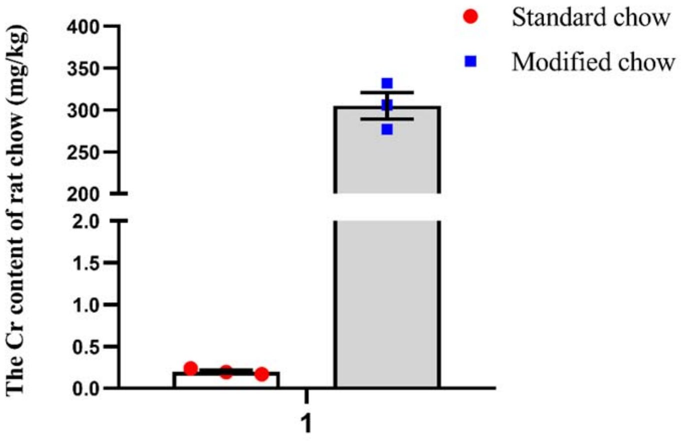

Supplementary Fig. 2 The concentration of Cr content in the standard commercial rodent chow and the modified rodent chow with Cr(VI) added. Values are mean ± SEM (n=3 per group).

(2) Results. The clarity of figures should be improved. I think that the authors should consider using another data visualisation strategy. It is very misleading to present plots where the spread of the data cannot be assessed, especially for experiments performed with a low number of replicates. The authors could use a dot plot representation which has become the standard to represent effect sizes in biology (https://journals.plos.org/plosbiology/article?id=10.1371/journal.pbio.1002128)

Response: Thanks for the comment. After careful reading of the paper recommended [9] and recent publications in Communications Biology [10,11], we have improved all the figures from bar graph to scatterplots within this article (**Fig.1B-C; Fig.2A-D,F; Fig.3B,D; Fig.4A-D; and Fig.5B-C**) and its Supplementary Information (**Supplementary Fig.1-3，6**). Please see the revised

manuscript and its Supplementary Information.

(3) I know by experience that the intragroup variation can be quite large in this type of gut microbiome experimental design. Could you provide dot plots to assess the effect size and the experimental variations for the most important genes having their expression altered in your study.

Response: Thanks for your comment. We have supplemented a figure (**Supplementary Fig. 5**) showing the genes whose expression were most altered in the revised Supplementary Information. As the gut microbiome experiment was performed by mixing feces samples from three individual mice in each of the four groups, only one dot is represented in each group. To confirm the expression alteration of some genes obtained from metatranscriptomic data, we have also performed quantitative RT PCR and supplemented the data (**Supplementary Fig. 6**). The qPCR results showed that the transcripts of three up-regulated DGEs comp53028, 53032 and 53086 in BT36+Cr(VI) group were on average 712.416, 245.242, and 226.731 times more than those in Cr(VI) group, respectively. In the Cr(VI) and control group, the selected genes (Ct value over 38) could hardly be detected. The results were in accordance with that observed in the metatranscriptomes analysis.

Please see the figure as follows:

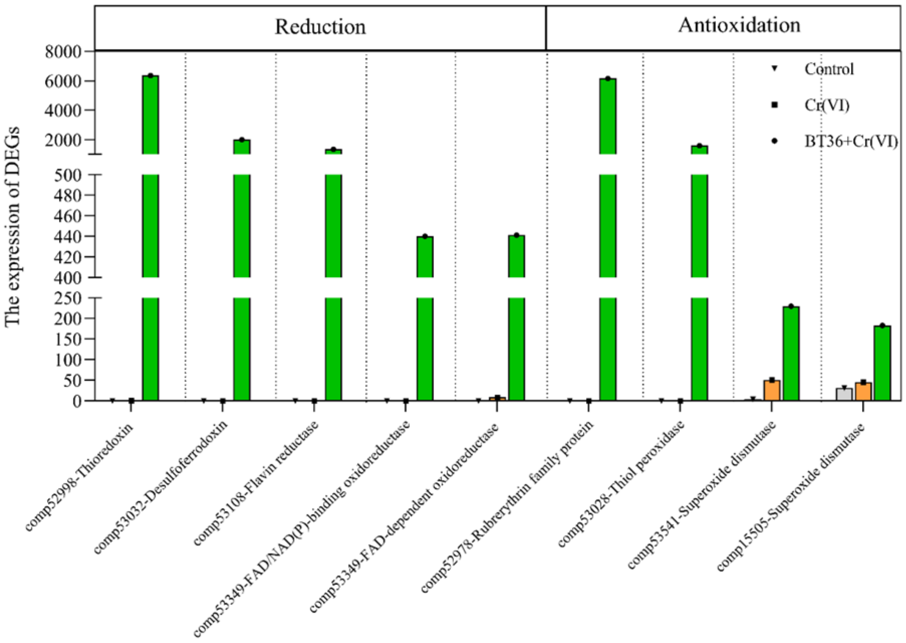

Supplementary Fig. 5 The expression of the most important genes related to Cr(VI) reduction and antioxidation.

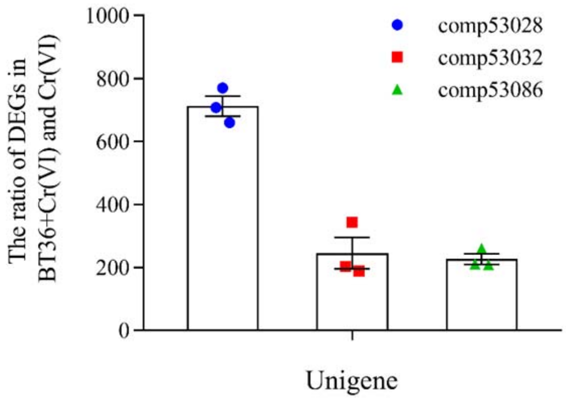

Supplementary Fig. 6 qPCR analysis of the expression profiles of DEGs in the gut microbiota of mice.

(4) The composition of the gut microbiome samples has been studied at the family and genus levels. Tools are available to perform the analysis at the species levels. It appears that the authors have used the QIIME Pipeline v. 1.7.0 which appears to be outdated. I would recommend to use a more recent method including the determination of amplicon sequence variants which have been recommended to replace OTUs and are used in more recent version of QIIME2.

Response: Thanks for your suggestion. We carefully studied the related analytical methods [12-14] and were assisted by professional analysts (Genesky Biotechnologies Inc., Shanghai) to reanalyze the 16S rRNA sequencing using QIIME2 and we have rewritten the method (**page 27 lines 561-581**), results (**page 9 lines 196-200**) and discussion (**page 20 lines 372-377**).

(5) It is always very convenient to have estimations of the number of reads and the quality of a gut microbiome study. Could you indicate how many reads per sample were sequenced and provide more details on the quality of this dataset (both for the

metatranscriptomics and the 16S analysis). In general, the bioinformatics pipeline is poorly described and more details could be useful.

Response: Thanks for your comment. We have added detailed description of the bioinformatics pipeline (**page 27 lines 561-581**) and the quality of the 16S analysis (**page 9 lines 196-200**) as well as the metatranscriptomics analysis (**pages 10-11 lines 222-233**) in the revised manuscript.

Please see the revised version as follows:

Method of "DNA extraction and processing for sequencing": 'The fresh feces excreted by mice in sterile cages were immediately collected in 2 mL sterile centrifuge tubes. Total bacterial DNA was isolated from fecal samples following the kit instructions (Tiangen Biotech (Beijing) Co., Ltd, DP328). Extracted DNA was used as a template for PCR amplification of bacterial 16S rRNA genes. PCR conditions, library preparation, and sequencing were performed as described previously [15]. Bacterial diversity was studied by Illumina MiSeq sequencing of the amplified V4-V5 region of 16S rRNA. The resulting sequence read files were carried out by using QIIME 2 pipeline (Quantitative Insights Into Microbial Ecology; http://qiime2.org) and its plugins. Specifically, the 'demux' plugin (https://github.com/qiime2/q2-demux) was used for the import of the demultiplexed paired-end sequencing reads and the creation of the 'artifact' file (i.e. qiime2 data format required for subsequent analyses). Further, the 'DADA2′ plugin [16] was applied for quality filtering (--p-max-ee 2, --p-trunk-q 2), chimera filtering ('consensus'), to trim primers (--p-trim-left-f 23, --p-trim-left-r 20), to truncate forward and reverse reads (--p-trunc-len-f 200, --p-trunc-len-r 200), and finally to collapse reads into amplicon sequence variants (ASVs). Samples were rarefied to 28,677 reads per sample for subsequent analysis to reduce the bias due to different sequencing depths. Taxonomy to these ASVs was assigned against the Greengenes database (version 13_8) by using the 'feature-classifier' plugin (https://github.com/qiime2/q2-feature-classifier) with the 'fit-classifier-sklearn'. Furthermore, we constructed the phylogenetic tree using muscle and Fast Tree 2 [17]. '

Results: ‘The sequencing of gut microbial 16S rRNA from four groups (n=12) resulted in a total of 1,263,860 reads. After quality filtering, 1,150,012 (min: 28,677; max: 290,528; mean: 95,834; median: 70,228) reads remained for the assignment of ASVs. Overall, 344,124 reads from 12 samples (average of 28,677 reads per sample) on the rarified data set were used for the further analysis. For the metatranscriptomics analysis, on average, 24 million raw sequence reads were obtained from the metatranscriptome of the four samples. After removing the adapters and low-quality reads, we obtained 60,546,020 clean reads with 8.17 G clean bases, 55,070,192 clean reads with 7.47 G clean bases, 44,050,042 cleans reads with 5.89 G clean bases and 40,869,144 clean reads with 5.46 G clean bases from BT36, BT36+Cr(VI), control and Cr(VI) group, respectively. The results indicated that the amount of data met the quality requirements for subsequent analysis. The results of the de novo assembly were shown in **Supplementary Table 6**. We obtained 14659, 78611, 87739 and 92503 unigenes with the N50 length of 556 bp, 813 bp, 945 bp, 738bp from BT36, BT36+Cr(VI), control and Cr(VI) group, respectively. There were most unigenes with length of 1-700 bp in all samples (**Supplementary Fig. 4**).’

(6) Line 519. ‘According to the manufacturer’ s instructions” Could you explain briefly what was the different methods

Response: Thanks for your comment. We have added a brief description of biochemical analysis of tissues in the Methods. Please see **page 26 lines 535-549**.

Supplemented content is as follows:

‘Frozen tissues were weighed and proportionally (w/v, 1/9) immersed in ice-cold 0.9% (w/v) NaCl, and then disrupted using a hand hold homogenizer. After centrifugated at 3000 × g at 4 ^{o}C for 10 min, suspension was obtained to determine the levels of malondialdehyde (MDA), glutathione peroxidase (GSH-PX), glutamic oxalacetic transaminase (AST), and catalase (CAT) in the liver and the levels of tumor necrosis factor-α (TNF-α) in the small intestine (Jiancheng Bioengineering Institute, Nanjing, China). The analysis was performed according to the manufacturer’s instructions. MDA can react with thiobarbituric acid to form a colored complex that has a

maximum absorbance at 532 nm and results were expressed as nmol/mg protein. GSH-Px activity was determined by measuring the decrease of GSH per min on the base of its catalysis. One unit of the enzyme activity was defined as a decrease of 1 μmol/L GSH per min for 1 mg tissue protein and the results were expressed as U/mg protein. CAT activity was determined by the decrease of the H_2O_2 at 240 nm and expressed as U/mg protein. TNF-α were measured using ELISA. Total protein concentration was determined by the Coomassie protein assay.'

(7) Line 572. There is a typo; 'flod-change'

Response: Thanks. We have corrected it as 'fold-change' on **page 29 line 629**.

(8) It is not clear to me if the authors have used faecal samples collected in the cage or directly from the animals during autopsy. If this is the latter, could you explain which sections of the gastrointestinal tract were sampled. The microbiome can be very different depending on the location of the sampling.

Response: Thanks for your comment. We agree with you that the microbiota composition can vary greatly depending the location. In this study, we used fresh faecal samples collected in sterile cages [18], which is a commonly accepted sample-collecting method in investigation of gut microbiome of both animals [19,20] and humans [21,22].

(9) Could you provide indications on the qualities of your transcript assemblies?

Response: Thanks for your comment. N50 or N90 can accurately represent the qualities of transcript assemblies. We have added these indications in the revised manuscript (**pages 10-11 lines 229-233**) and Supplementary Information.

Please see the content as follows:

Supplementary Table 6. Statistics of assembly result.

**Sample**	**Seqs**	**Seqs bases (bp)**	**N50 (bp)**	**N90 (bp)**	**Max (bp)**	**Min (bp)**

BT36	14659	8174450	556	329	9575	300
BT36+Cr(VI)	78611	55770480	813	347	29842	300
Control	87739	67965177	945	358	28959	300
Cr(VI)	92503	61891539	738	344	28282	300

Seqs: The number of sequences in a transcript; Seq bases: The total sequence length of a transcript; N50（N90）: Sequence the transcripts in order of length, scan the length of each sequence one by one from the largest to the smallest, and accumulate. When the accumulated value exceeds 50% (90%) of the total length of all sequences for the first time, the length value of the scanned sequence is N50 (N90); Compared with the average length of the sequence, N50 (N90) can more accurately represent the splicing effect of sequence. Max: The sequence length of the longest transcript; Min: The sequence length of the shortest transcript.

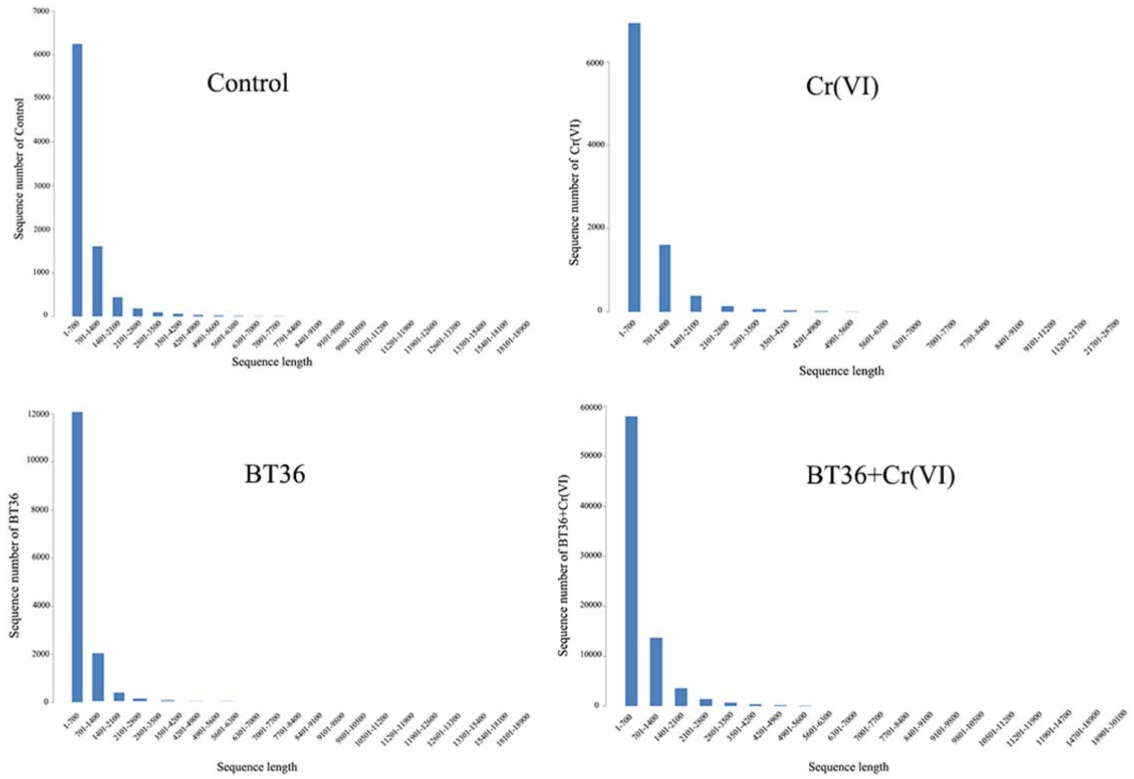

Supplementary Fig 4. The sequence length distribution of four groups.

“On average, 24 million raw sequence reads were obtained from the

metatranscriptome of the four samples. After removing the adapters and low-quality reads, we obtained 60,546,020 clean reads with 8.17 G clean bases, 55,070,192 clean reads with 7.47 G clean bases, 44,050,042 cleans reads with 5.89 G clean bases and 40,869,144 clean reads with 5.46 G clean bases from BT36, BT36+Cr(VI), control and Cr(VI) group, respectively. The results indicated that the amount of data met the quality requirements for subsequent analysis. The results of the *de novo* assembly were shown in **Supplementary Table 6**. We obtained 14659, 78611, 87739 and 92503 unigenes with the N50 length of 556 bp, 813 bp, 945 bp, 738bp from BT36, BT36+Cr(VI), control and Cr(VI) group, respectively. **Supplementary figure 4** presented the sequence length distribution. There were most unigenes with length of 1-700 bp in all samples."

(10) I suggest to cite the most recent relevant references on the topic which have been performed in laboratory animals such as https://www.ncbi.nlm.nih.gov/pubmed/31377140

Response: Thanks for your suggestion. We have cited the suggested reference in discussion on **page 19 line 339**.

Revised version as follows:

'In this study, exposure to Cr(VI) induced significant oxidative stress in the liver of mice, as indicated by the increased Cr contents and MDA levels, which was consistent with a previous study using rats exposed to Cr (VI) [23]. '

References:

1 Tian, F. *et al.* Lactobacillus plantarum CCFM8661 alleviates lead toxicity in mice. *Biological trace element research* **150**, 264-271 (2012).

2 Zhai, Q. *et al.* Protective effects of Lactobacillus plantarum CCFM8610 against chronic cadmium toxicity in mice indicate routes of protection besides intestinal sequestration. *Appl. Environ. Microbiol.* **80**, 4063-4071 (2014).

3 Zhai, Q. *et al.* Oral administration of probiotics inhibits absorption of the heavy metal cadmium by protecting the intestinal barrier. *Appl. Environ. Microbiol.* **82**, 4429-4440 (2016).

4 Zhai, Q. *et al.* Effects of subchronic oral toxic metal exposure on the intestinal microbiota of mice. *Science Bulletin* **62**, 831-840, doi:https://doi.org/10.1016/j.scib.2017.01.031 (2017).

5 Richardson, J. B. *et al.* Exposure to toxic metals triggers unique responses from the rat gut microbiota. *Scientific Reports* **8**, 6578, doi:10.1038/s41598-018-24931-w (2018).

6 Xia, J. *et al.* Chronic exposure to low concentrations of lead induces metabolic disorder and dysbiosis of the gut microbiota in mice. *Science of The Total Environment* **631-632**, 439-448, doi:https://doi.org/10.1016/j.scitotenv.2018.03.053 (2018).

7 Breton, J. *et al.* Ecotoxicology inside the gut: impact of heavy metals on the mouse microbiome. *BMC Pharmacology and Toxicology* **14**, 62, doi:10.1186/2050-6511-14-62 (2013).

8 GB2762-2017. National Food Safety Standard Maximum Levels of Contaminants in Foods. China Food and Drug Administration, Beijing. (2017).

9 Weissgerber, T. L., Milic, N. M., Winham, S. J. & Garovic, V. D. Beyond Bar and Line Graphs: Time for a New Data Presentation Paradigm. *PLOS Biology* **13**, e1002128, doi:10.1371/journal.pbio.1002128 (2015).

10 Zhang, Z. *et al.* Ability of prebiotic polysaccharides to activate a HIF1α-antimicrobial peptide axis determines liver injury risk in zebrafish. *Communications Biology* **2**, 274, doi:10.1038/s42003-019-0526-z (2019).

11 Kaliannan, K. *et al.* Multi-omic analysis in transgenic mice implicates omega-6/omega-3 fatty acid imbalance as a risk factor for chronic disease. *Communications Biology* **2**, 276, doi:10.1038/s42003-019-0521-4 (2019).

12 Hernández, M. *et al.* Fecal Microbiota of Toxigenic Clostridioides difficile-Associated Diarrhea. *Front Microbiol* **9**, 3331-3331, doi:10.3389/fmicb.2018.03331 (2019).

13 Šrut, M., Menke, S., Höckner, M. & Sommer, S. Earthworms and cadmium – Heavy metal resistant gut bacteria as indicators for heavy metal pollution in soils? *Ecotoxicology and Environmental Safety* **171**, 843-853, doi:https://doi.org/10.1016/j.ecoenv.2018.12.102 (2019).

14 Bolyen, E. *et al.* Reproducible, interactive, scalable and extensible microbiome data science using QIIME 2. *Nature Biotechnology* **37**, 852-857, doi:10.1038/s41587-019-0209-9 (2019).

15 Wu, G. *et al.* Gut remediation: a potential approach to reducing chromium accumulation using Lactobacillus plantarum TW1-1. *Scientific reports* **7**, 15000, doi:https://doi.org/10.1038/s41598-017-15216-9 (2017).

16 Callahan, B. J. *et al.* DADA2: High-resolution sample inference from Illumina amplicon data. *Nature Methods* **13**, 581-583, doi:10.1038/nmeth.3869 (2016).

17 Price, M. N., Dehal, P. S. & Arkin, A. P. FastTree 2 – Approximately Maximum-Likelihood Trees for Large Alignments. *PLOS ONE* **5**, e9490, doi:10.1371/journal.pone.0009490 (2010).

18 Chassaing, B., Aitken, J. D., Malleshappa, M. & Vijay-Kumar, M. Dextran sulfate sodium (DSS)-induced colitis in mice. *Current protocols in immunology* **104**, 15 25 11-15 25 14, doi:10.1002/0471142735.im1525s104 (2014).

19 Ntemiri, A. *et al.* Retention of Microbiota Diversity by Lactose-Free Milk in a Mouse Model of Elderly Gut Microbiota. *Journal of Agricultural and Food Chemistry* **67**, 2098-2112, doi:10.1021/acs.jafc.8b06414 (2019).

20 Velázquez, K. T. *et al.* Prolonged high-fat-diet feeding promotes non-alcoholic fatty liver disease and alters gut microbiota in mice. *World J Hepatol* **11**, 619-637, doi:10.4254/wjh.v11.i8.619 (2019).

21 Yano, Jessica M. *et al.* Indigenous Bacteria from the Gut Microbiota Regulate Host Serotonin

Biosynthesis. *Cell* **161**, 264-276, doi:https://doi.org/10.1016/j.cell.2015.02.047 (2015).

22 Jiang, H. *et al.* Altered fecal microbiota composition in patients with major depressive disorder. *Brain, Behavior, and Immunity* **48**, 186-194, doi:https://doi.org/10.1016/j.bbi.2015.03.016 (2015).

23 Karaulov, A. V. *et al.* Long-term effects of chromium on morphological and immunological parameters of Wistar rats. *Food and Chemical Toxicology* **133**, 110748, doi:https://doi.org/10.1016/j.fct.2019.110748 (2019).

REVIEWERS' COMMENTS:

Reviewer #1 (Remarks to the Author):

The authors have addressed all my concerns. I think this manuscript is qualified for publication.
